# “Carrying the burden silently”: A mixed-methods analysis of common mental health disorders among women caregivers living with HIV in Enugu, Nigeria

**DOI:** 10.1371/journal.pmen.0000764

**Published:** 2026-10-23

**Authors:** Ugochukwu Uzoechina Nwokoro, Ifeyinwa Lilian Ezenwosu, Elias Chikee Aniwada, Izuchukwu Frank Obi, Justus Uchenna Onu, Chidiebere Clifford Aniagboso, Eunice Enabulele, Chinyere Nkiruka Okafor, Chijioke Thaddeus Asogwa, John Olajide Olawepo, Chika N. Onwasigwe

**Affiliations:** 1 Department of Community Medicine, Federal Teaching Hospital, Owerri, Imo State, Nigeria; 2 Department of Community Medicine, University of Nigeria Teaching Hospital, Enugu, Enugu State, Nigeria; 3 Department of Community Medicine, Faculty of Medical Sciences, College of Medicine, University of Nigeria Enugu Campus, Enugu, Enugu State, Nigeria; 4 Department of Mental Health, Nnamdi Azikiwe University, Awka, Anambra State, Nigeria; 5 Department of Community Medicine, Enugu State University College of Medicine, Parklane, Enugu, Enugu State, Nigeria; 6 Department of Public Health and Health Sciences, Bouve College of Health Sciences, Northeastern University, Boston, Massachusetts, United States of America; 7 IVAN Research Institute, University of Nigeria, Old UNTH, Enugu, Nigeria; Marie Adelaide Leprosy Center, PAKISTAN

## Abstract

Women caregivers living with HIV (WCLHIV) face a dual burden of managing their own health while caring for their families, increasing their vulnerability to mental health disorders. This study aimed to ascertain the prevalence and factors associated with common mental health disorders among WCLHIV in Enugu, Nigeria. A cross-sectional study employing a mixed method approach was conducted among 1,011 WCLHIV accessing anti-retroviral therapy in four tertiary health facilities in Enugu State, Nigeria. Quantitative data were obtained face-to-face using pre-tested, validated tools to assess the prevalence of depression, anxiety, stigma, and other psychological characteristics. Multivariate logistic regression identified significant risk factors of mental health disorders at a 95% confidence interval and p < 0.05. Four focus group discussions involving 35 WCLHIV explored factors impacting their mental well-being. Qualitative data were analyzed thematically using an inductive approach. Participants had a mean age of 44.3 (SD ± 8.44) years. The prevalence of depression and anxiety was 25.7% and 19.2%, respectively, while 79.0% reported high perceived stigma. Having primary education (AOR = 1.53; 95% CI: 1.06 –2.22), and low social support (AOR = 2.32; 95% CI: 1.11–5.07) were risk factors while low income (AOR = 0.42; 95% CI: 0.21–0.85), and low perceived stress (AOR = 0.05; 95% CI: 0.02–0.15) were protective of depression. Low perceived stress was protective of anxiety (AOR = 0.07; 95% CI: 0.02–0.22). Qualitative findings converged with quantitative data, highlighting the impact of stigma, poor social support, pill burden, fear of status disclosure, and financial strain on the mental health of WCLHIV.The prevalence of common mental health disorders was high among WCLHIV with a substantial proportion experiencing depression and anxiety, reflecting the significant impact of stigma, low social support, and stress. Targeted multi-level psychosocial interventions and community empowerment programs are needed to strengthen social support and reduce stigma among WCLHIV.

## Introduction

Globally, HIV remains a major public health challenge, disproportionately affecting women. In 2024, an estimated 40.8 million people were living with HIV, with women accounting for more than 53% of this population [1]. Approximately, 3,300 out of the estimated 4,000 new cases of HIV infections that occur globally each week among young women are reported in sub-Saharan Africa [1]. In sub-Saharan Africa, women account for over 63% of HIV infections and have more than twice the likelihood of living with HIV compared to their male counterparts [1,2].

Globally, the prevalence of mental health disorders among people living with HIV (PLWH) remains high, estimated at 28% to 62%, with higher rates reported in sub-Saharan Africa (SSA) [3]. Women living with HIV (WLHIV) bear a particularly heavy burden, experiencing significantly higher rates of mental health challenges than their male counterparts [4,5]. Among WLHIV, anxiety and depression are the most frequently reported conditions, with pooled prevalence estimates ranging from 5.9% to 61% for depression and 28.9% to 61% for anxiety in SSA [6]. The wide variation in prevalence estimates reported in the systematic review reflects differences in study populations, geographical and sociocultural contexts, as well as methodological approaches to assessing depression and anxiety across studies [6].

Beyond HIV-related stressors, gender may influence the mental health of women living with HIV through socially defined roles and inequalities, including expectations around economic dependence, unequal relationship dynamics, reduced decision-making power, and self-sacrificing roles that prioritize the needs of others over their own [7]. These gender-related pressures are not limited to women with caregiving responsibilities, as traditional gender roles have been associated with poorer mental health among women more broadly [7]. However, for women who also have caregiving responsibilities, meeting the physical, emotional, and practical needs of children and other family members places additional demands on women already experiencing gender- and HIV-related stressors [4].

The burden of informal caregiving often lies on female family caregivers, reflecting social expectations that women assume nurturing and caregiving roles, which increase their vulnerability to anxiety and depression [8]. This disproportionate impact of the two common mental health disorders can be due to stressors arising from women’s responsibilities that extend beyond reproductive functions to include household management, work-related obligations, financial responsibilities, and maintaining family and social relationships [4]. Evidence has shown that women experience 3–5 folds more mental health issues after HIV diagnosis compared to before the diagnosis [9]. As frequent users of healthcare services for antiretroviral treatment, they often face the dual burden of managing their health conditions while fulfilling caregivers’ responsibilities [10]. Furthermore, the fear of rejection and stigma associated with disclosure of their HIV status can negatively affect their mental health and the children’s social development [11]. The negative effects of their mental health ultimately impact the family, influencing partner relationships, sibling dynamics, and the overall family environment [4].

Anxiety and depression are long-standing public health challenges that often go unnoticed among women caregivers living with HIV, who carry caregiving responsibilities throughout their lives [12]. This can affect their medication adherence, causing loss to follow-up and poor life expectancy if not addressed [12]. Although several studies have assessed the common mental health disorders of caregivers caring for people living with HIV, there is a limited study on the burden and drivers of common mental health challenges of women caregivers who are living with HIV and are faced with the double burden of taking care of their health challenges as well as their caregiving roles in the society.

Thus, understanding the prevalence and context-specific factors associated with common mental health disorders among WCLHIV is important for addressing the mental health burden and the determinants of mental health in this sub-population. Furthermore, this study provides evidence that policymakers and HIV program managers need to implement focused interventions that will promote, protect, and restore the mental health of women caregivers living with HIV. This study aimed to ascertain the prevalence and factors associated with common mental health disorders among women caregivers living with HIV in Enugu, Nigeria

## Materials and methods

### Ethics statement

Ethical approval for this study was obtained from the Health Research Ethics Committee of the University of Nigeria Teaching Hospital, Ituku-Ozalla, Enugu (UNTH/HREC/2024/04/939).

Written informed consent was obtained from all study participants after providing detailed information on the study objective, procedure, anticipated risks and benefits as well as their rights to refuse to participate. Confidentiality of all study participants was ensured and maintained during and after the study by ensuring the anonymity of study participants. Participants who agreed to take part signed the informed consent form. For participants who were unable to read or write, the consent form was read aloud in the presence of an impartial witness, the participant thumb printed the form, and the witness signed.

### Study setting

The study was conducted in four HIV clinics, which include two government-owned teaching hospitals (University of Nigeria Teaching Hospital (UNTH), Enugu State University of Science and Technology Teaching Hospital (ESUTH)), and two missionary hospitals (Annunciation Specialist Hospital and Mother of Christ Specialist Hospital) in Enugu State, Nigeria. These four facilities serve as referral centres for other health facilities in Enugu and in Southeast Nigeria. They provide promotive, preventive, and treatment services to women caregivers living with HIV. In line with the World Health Organization HIV treatment guidelines, health facilities in the country implement the “test and treat” strategy, all women caregivers living with HIV in the selected health facilities are on antiretroviral therapy [13].

### Study design

We employed a mixed-methods convergent parallel design, combining a facility-based survey with focus group discussions which was conducted simultaneously. This approach enabled a comprehensive understanding of the factors contributing to mental health disorders among women caregivers living with HIV and allowed for triangulation of the findings. Because different participants contributed to the quantitative and qualitative components, integration occurred at the level of themes rather than through within-participant comparison. We reported the quantitative component following the Strengthening the Reporting of Observational Studies in Epidemiology (STROBE) guidelines, and the qualitative component following the Consolidated Criteria for Reporting Qualitative Research (COREQ) checklist. The completed STROBE and COREQ checklists are provided as S1 and S2 Checklists, respectively.

### Study population, sample size and participants’ recruitment

The study included women caregivers living with HIV aged ≥18 years who have a child and are directly involved with the care of the child, provided informed consent and are accessing HIV care in the four HIV clinics within the tertiary health facilities. WCLHIV who were too ill to participate or on treatment for a mental disorder were excluded. The recruitment of participants started from 2^nd^ May 2024 and ended 28^th^ October 2024

In the quantitative phase of this study, the minimum sample size was obtained using the formula for a single population proportion formula, considering the following: 22.7% as the proportion of respondents with anxiety and depression [12], a 95% confidence level (α = 0.05), and a margin of error of 5%. A further 10% was added to account for non-response, yielding a minimum sample size of 300. However, all women who met the inclusion criteria within the period of the study were recruited, resulting to a total of 1,011 WCLHIV.

In the qualitative phase, eligible WCLHIV were purposively selected for focus group discussions (FGDs). To minimize respondent fatigue and ensure diverse perspectives, only those who had not participated in the quantitative survey were included in the phase.

### Quantitative data collection

Data were collected in the HIV clinics using a pre-tested, structured, interviewer-administered questionnaire. This method of administration was used to accommodate participants with limited literacy. Where participants had difficulty understanding questions in English, trained research assistants explained the items in the participant’s preferred language while maintaining their intended meaning. The questionnaire was pre-tested among women living with HIV in a comparable HIV clinic setting to assess the clarity, comprehension, cultural appropriateness, and feasibility before the main data collection. The interviews were conducted by trained research assistants, who were nurses and counsellors providing HIV care. The questionnaire consisted of six standardized tools. The instruments used in this study have demonstrated acceptable psychometric properties in Nigerian populations. The PHQ-9 and GAD-7 have been validated in Nigerian populations [14,15], the MSPSS and 12-item Zarit Burden Interview have also demonstrated acceptable reliability and validity in Nigerian settings [16,17]. The PSS-10 has been previously used among Nigerian women living with HIV and demonstrated good internal consistency [18]. The HIV Stigma Scale has similarly been used and evaluated among people living with HIV in comparable settings [19]. Internal consistency of the multi-item instruments was assessed in the present study using Cronbach’s alpha. The PHQ-9 (α = 0.874), GAD-7 (α = 0.818), MSPSS (α = 0.876), PSS-10 (α = 0.875), and 12-item Zarit Burden Interview (α = 0.752) demonstrated acceptable to good internal consistency, while the 12-item HIV Stigma Scale showed more modest internal consistency (α = 0.634).

***Patient Health Questionnaire-9 (PHQ-9):*** The PHQ-9 [20] was used to assess depression. It comprises 9 items rated on a four-point Likert scale ranging from “not at all” (0) to “most days” [3], assessing the frequency of depressive symptoms over the preceding two weeks. The total score ranges from 0 to 27. Respondents with scores ≥10 were categorized as having depression, while those with scores <10 were categorized as not depressed.

***Generalized Anxiety Disorder-7 (GAD-7):*** The GAD-7 [21] was used to assess anxiety disorders. It consists of 7 items rated on a four-point scale (0 = “not at all,” 1 = “several days,” 2 = “more than half the days,” 3 = “nearly every day”). Scores range from 0 to 21. Respondents with scores ≥10 were categorized as having an anxiety disorder, while those with scores <10 were categorized as not having an anxiety disorder.

***Perceived Stress Scale-10 (PSS-10):*** The PSS-10 [22] measured perceived stress by assessing how unpredictable, uncontrollable, and overwhelming situations were in the past month. It consists of 10 items rated on a five-point scale (0 = “never” to 4 = “very often”), with total scores ranging from 0 to 40. Stress levels were categorized as: mild stress (0–13), moderate stress [14–26], and high stress [27–40].

***HIV Stigma Scale:*** This 12-item tool [23] assessed stigma across four subscales: personalized stigma, disclosure concerns, public attitudes, and negative self-image. Each item was rated on a four-point Likert scale (1 = strongly disagree to 4 = strongly agree). Total scores <30 indicated low stigma, while scores ≥30 indicated high stigma.

***Multidimensional Scale of Perceived Social Support (MSPSS):*** The MSPSS [24] measured perceived social support from three sources: family, friends, and a significant other. It contains 12 items rated on a seven-point Likert scale (1 = “very strongly disagree” to 7 = “very strongly agree”). Total scores range from 12 to 84, categorized as low support (12–35), medium support (36–60), and high support (61–84).

***Zarit Burden Interview (ZBI):*** The ZBI, a 12-item tool [25], assessed caregiver burden using a five-point Likert scale (0 = never to 4 = nearly always). Scores were categorized as follows: no to mild burden (0–10), mild to moderate burden [11–20], and high burden (> [20].

### Qualitative data collection

Two eligible women declined participation in the FGDs because they felt that participation would take too much of their time. Eligible participants were approached face-to-face at the HIV clinics and invited to participate in the FGDs. Focus group discussions (FGDs) were conducted among WCLHIV in the HIV clinics, consisting of 8–10 FGD participants per group; a total of four FGDs involving 35 participants were conducted using the English language. An FGD guide developed by the authors was used to elicit information from FGD participants regarding stressors to mental health disorders. Each FGD lasted for about 65 minutes and was moderated by UUN and ILE, male and female public health physicians, respectively, both with experience in qualitative research and fellows of the West African College of Physicians. No repeat interviews or FGDs were conducted. No prior relationship existed between the FGD moderators and participants before data collection. Participants were informed that the moderators were members of the research team and were conducting the study to understand factors affecting the mental health of women caregivers living with HIV. No specific assumptions or personal interests were formally documented before data collection. All FGDs were audio-recorded, and a trained research assistant took field notes. At the end of each interview, a summary of findings and observations was compiled by the moderator and the note-taker. Audio files were transcribed verbatim immediately after the interview.

### Quantitative data analysis

The dependent variables were depression and anxiety disorders. Data were cleaned and entered into the International Business Machine-Statistical Product and Service Solutions (IBM-SPSS) Version 20.0. Means and standard deviations of numeric variables were calculated, while frequencies and proportions were generated for categorical variables. Chi-square test was used to compare proportions as well as measure associations between categorical variables.

Logistic regression analysis was performed to determine predictors of depression and Anxiety disorders for variables that showed significant association (p-value <0.2) with depression and Anxiety disorders at the bivariate level. The level of statistical significance was set at p-value <0.05 for all cross-tabulations and inferential analysis; a 95% confidence interval was used in the data presentation for the regression model.

### Qualitative data analysis

For quality assurance purposes, the scripts were compared with the written notes for completeness and accuracy by the research team during review meetings. Then each script was checked against the audiotape by an independent reviewer. Furthermore, the interview audiotapes were doubly transcribed after which both scripts were checked for similarity and where differences existed, these were reconciled by the transcribers. The emerging and predetermined approach was employed in this study. The themes from the FGDs were reviewed by the authors, after which the various sub-themes were grouped under four major themes. QDA Miner Lite v2.0.6 was used in the analysis.

## Results

A total of 1,024 women were approached across the four study facilities. Three were excluded because they did not meet the eligibility criteria; two were critically ill and one was receiving treatment for a mental health disorder. Ten women declined participation. A total of 1,011 women were included in the final analysis. Participant recruitment and flow by study facility are shown in Fig 1.

**Fig 1:**
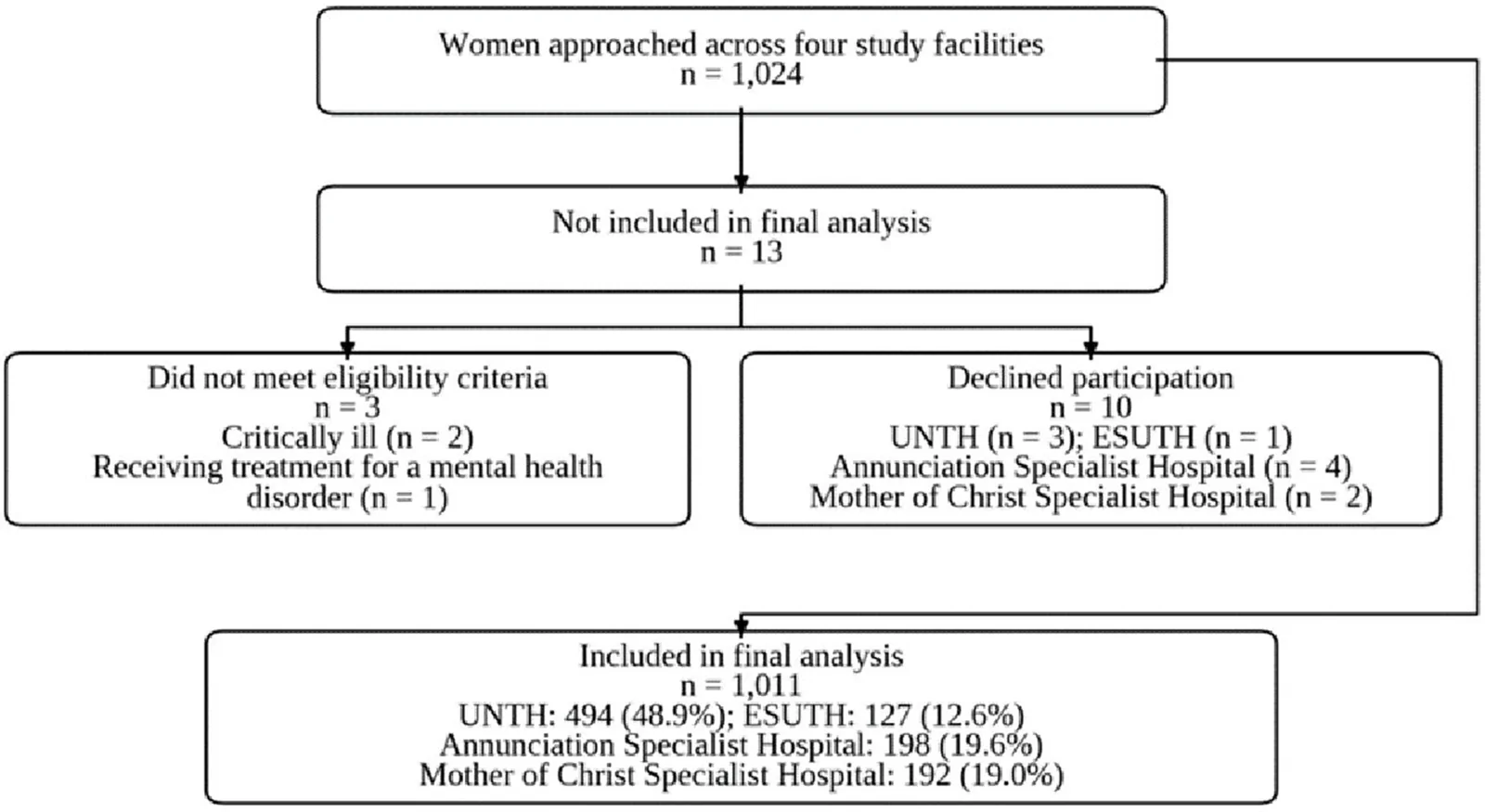
Flow diagram of participant recruitment and inclusion.

### Quantitative findings

#### Socio-demographic characteristics of the women caregivers living with HIV.

Table 1 shows the socio-demographic characteristics of the respondents. A total of 1,011 WCLHIV participated in the study with a mean age of 44.3 ± 8.44 years. The majority were married (60.2%), had attained secondary education or higher (73.2%), and were employed (98.3%). Among married respondents, 97.7% of spouses were employed, and 78.5% had attained secondary education or above. Over half of the WCLHIV (52.2%) had three or more children, and about 10.9% were caregivers to a child living with HIV. Most respondents (83.9%) knew their spouse’s HIV status, with nearly half (47.6%) reporting HIV-positive partners and most households (65.8%) earned a monthly income of below ₦50,000.

**Table 1:** Socio-demographic characteristics of the women caregivers living with HIV.

Variables	Frequency (n = 1011)	Percentage
**Age of the respondents (years)**		
18 – 49	750	74.2
≥50	261	25.8
Mean Age ± SD	44.3 ± 8.44	
**Marital Status**		
Married	609	60.2
Widowed	310	30.7
Single	92	9.1
**Educational attainment of the respondents**		
No formal education	48	4.7
Primary	223	22.1
Secondary and above	740	73.2
**Employment status of the respondents**		
Unemployed	17	1.7
Employed	994	98.3
**Employment status of the spouse (n = 609)**		
Unemployed	14	2.3
Employed	595	97.7
**Educational attainment of the spouse (n = 609)**		
No formal education	24	3.9
Primary	107	17.6
Secondary and above	478	78.5
**Number of Children by the respondents**		
0	11	1.1
1 – 2	472	46.7
≥3	528	52.2
Mean ± SD	2.83 ± 1.58	
**Caregiver to a child living with HIV**		
Yes	110	10.9
No	901	89.1
**Number of children living with HIV (110)**		
1	100	90.9
2	7	6.4
3	3	2.7
Mean±SD	1.12 ± 0.41	
**Age of children living with HIV (years)**		
<10	35	31.8
≥10	75	68.2
Mean±SD	12.41 ± 5.71	
**Relationship to the child (n = 110)**		
Mother	105	95.5
Aunty	5	4.5
**Do you know the HIV status of spouse?**		
Yes	511	83.9
No	98	16.1
**HIV status of the partner (n = 511)**		
Positive	243	47.6
Negative	268	52.4
**Household size**		
<5	543	53.7
≥5	468	46.3
Mean ± SD	4.49 ± 1.98	
**Family income (naira)**		
<50000	665	65.8
≥50000 – 99000	301	29.8
≥100000	45	4.5
Median ± IQR	30000 ± 30000	

#### Clinical characteristics of the women caregivers living with HIV.

The median duration since HIV diagnosis was 197.7 ± 108 months, with 64.8% living with HIV for 10 years or more and 63.9% had been on treatment for at least 10 years. Most respondents (96.8%) had a viral load <1000 copies/ml in the past year and only 24.6% reported any relationship with a health worker. The majority (87.0%) were on first-line ART, and nearly all (99.5%) were in WHO clinical stage 1. About 14.4% reported having a co-morbidity, mainly hypertension (87%), followed by diabetes (4.1%) and other minor conditions, as shown in Table 2.

**Table 2:** Clinical characteristics of the women caregivers living with HIV.

Variables	Frequency	Percent
**Duration of HIV since first Diagnosis (months)**		
<120	356	35.2
≥120	655	64.8
Median ± IQR	197.67 ± 108	
**Duration on ART (months)**		
<120	365	36.1
≥120	646	63.9
Median ± IQR	195.51 ± 108	
**Viral load (the past one year)**		
<1000	979	96.8
≥1000	32	3.2
Median ±IQR	3026.96 ± 19	
**Any personal relationship with a health worker**		
Yes	249	24.6
No	762	75.4
**Relationship with the Health worker (n = 249)**		
Siblings	37	14.9
Relatives	46	18.5
Friend	154	61.8
Child	8	3.2
Spouse	4	1.6
**Line of Drug**		
First line	880	87.0
Second line	131	13.0
**Current Clinical Stage**		
Stage 1	1006	99.5
Stage 2	5	0.5
**Do you have any co-morbidity**		
Yes	146	14.5
No	817	80.8
I don’t know	48	4.7
**Self-reported co-morbidities (n = 146)**		
Hypertension	127	87.0
Diabetes Mellitus	6	4.1
Peptic ulcer	5	3.4
Eye problem	4	2.7
Asthma	2	1.4
Arthritis	1	0.7
Cancer	1	0.7

#### Psychological characteristics of the respondents.

As shown in Table 3, most respondents reported moderate social support (71.1%), while 24.8% had high and 4.1% had low support. A majority (79.0%) experienced high perceived stigma. Most respondents (86.4%) reported moderate stress, with 9.5% experiencing low and 4.1% experiencing high stress. In terms of caregiver burden, 68.1% had a mild-to-moderate burden, and 22.7% had a high burden. Overall, 25.7% of respondents were depressed, while 19.2% had anxiety disorder.

**Table 3:** Psychological characteristics of the respondents.

Variables	Frequency	Percent
**Social Support**		
Low	41	4.1
Moderate	719	71.1
High	251	24.8
Mean±SD	53.06 ± 11.50	
**Perceived Stigma**		
Low stigma	212	21.0
High	799	79.0
Mean±SD	32.84 ± 4.04	
**Perceived Stress**		
Low stress	96	9.5
Moderate stress	874	86.4
High stress	41	4.1
Mean±SD	19.87 ± 4.04	
**Caregivers’ burden**		
No – mild burden	93	9.2
Mild – moderate	688	68.1
High burden	230	22.7
Mean±SD	16.52 ± 4.83	
**Common Mental Diseases**		
**Depression**		
Not depressed (PHQ score < 10)	751	74.3
Depressed (PHQ score ≥ 10)	260	25.7
Median ±IQR	2.00 ± 10.00	
**Anxiety**		
No Anxiety disorder (GAD Score < 10)	817	80.8
Anxiety disorder (GAD score ≥ 10)	194	19.2
Median±IOR	3.00 ± 7.00	

#### Factors associated with depression among WCLHIV.

At the bivariate level, marital status, educational attainment, household size, household income, presence of co-morbidity, social support, perceived stress, and caregiver burden were significantly associated with depression (p < 0.05). In the multivariate logistic regression analysis, primary education (AOR = 1.53; 95% CI: 1.06 – 2.22), household income below ₦50,000 (AOR = 0.42; 95% CI: 0.21 –0.85), low social support (AOR = 2.32; 95% CI: 1.11–5.07), and low stress (AOR = 0.05; 95% CI: 0.02–0.15) remained significant predictors of depression among WCLHIV (Table 4). The multivariable logistic regression model for depression showed no evidence of poor fit (Hosmer–Lemeshow χ^2^ = 11.70, df = 8, p = 0.165) and had a Nagelkerke R^2^ of 0.157.

**Table 4:** Factors associated with depression among women caregivers living with HIV.

Variables	(n = 1011) Depressed Not Depressed N (%) N (%)		χ2(p-value on bivariate analysis)	AOR in multivariate analysis (95% CI)
**Age of the respondents (years)**				
18 – 49	181 (24.1)	569 (75.9)	3.815 (0.051)	0.902 (0.628 – 1.294)
≥50	79 (30.3)	182 (69.7)		1.000
**Marital Status**				
Married	124 (20.4)	485 (79.6)	25.766 (<0.001)	0.820 (0.471 – 1.428)
Widowed	111 (35.8)	199 (64.2)		1.381(0.782 – 2.437)
Single	25 (27.2)	67 (72.8)		1.000
**Educational attainment of the respondents**				
No formal education	15 (31.3)	33 (68.8)	14.622 (0.001)	1.399 (0.691 – 2.832)
Primary	78 (35.0)	145 (65.0)		1.529 (1.056 – 2.216)*
Secondary and above	167 (22.6)	573 (77.4)		1.000
**Employment status**				
Unemployed	6 (35.3)	11 (64.7)	0.830 (0.401)	
Employed	254 (25.6)	740 (74.4)		
**Employment status of the spouse**				
Unemployed	7 (50.0)	7 (50.0)	7.763 (0.012)	
Employed	117 (19.7)	478 (80.3)		
**Level of education of the spouse**				
No formal education	3 (12.5)	21 (87.5)	2.624 (0.269)	
Primary	27 (25.2)	80 (74.8)		
Secondary and above	94 (19.7)	384 (80.3)		
**Personal relationship with Health worker**				
Yes	73 (29.3)	176 (70.7)	2.241 (0.134)	0.712 (0.507 – 1. 025)
No	187 (24.5)	575 (75.5)		1.000
**Number of living children**				
0	1 (9.1)	10 (90.9)	1.715 (0.424)	
1 – 2	120 (25.4)	352 (74.6)		
≥3	139 (26.3)	389 (73.7)		
**Currently providing care for a child living with HIV**				
Yes	32 (29.1)	78 (70.9)	0.735 (0.391)	
No	228 (25.3)	673 (74.7)		
**HIV status of the spouse**				
Positive	47 (19.3)	196 (80.7)	0.191 (0.662)	
Negative	56 (20.9)	212 (79.1)		
**Household size**				
<5	157 (28.9)	386 (71.1)	6.273 (0.012)	1.193 (0.868 – 1.639)
≥5	103 (22.0)	365 (78.0)		1.000
**Household income per month (Naira)**				
<50000	152 (22.9)	513 (77.1)	8.830 (0.012)	0.424 (0.212 – 0.845)*
50000 – 99000	92 (30.6)	209 (69.4)		0.618 (0.304 – 1.254)
≥100000	16 (35.6)	29 (64.4)		1.000
**Duration of living with HIV (months)**				
<120	89 (25.0)	267 (75.0)	0.148 (0.701)	
≥120	171 (26.1)	484 (73.9)		
**Duration on ART (Months)**				
<120	94 (25.8)	271 (74.2)	0.000 (0.984)	
≥120	166 (25.7)	480 (74.3)		
**Viral load (copies/ml)**				
<1000	251 (25.6)	728 (74.4)	0.100 (0.751)	
≥1000	9 (28.1)	23 (71.9)		
**Presence of other co-morbidities**				
Yes	47 (32.2)	99 (67.8)	6.843 (0.033)	1.610 (0.758 – 3.421)
No	196 (24.0)	621 (76.0)		0.756 (0.494 – 1.159)
I don’t know	17 (35.4)	31 (64.6)		1.000
**Social support**				
Low support	17 (41.5)	24 (58.5)	16.665 (<0.001)	2.321 (1.110 – 5.072)*
Moderate	200 (27.8)	519 (72.2)		1.866 (1.242 – 2.803)*
High	43 (17.1)	208 (82.9)		1.000
**Stigma**				
Low stigma	49 (23.1)	163 (76.9)	0.952 (0.329)	
High stigma	211 (26.4)	588 (73.6)		
**Stress**				
Low stress	7 (7.3)	89 (92.7)	51.644 (<0.001)	0.053 (0.019 – 0.150)*
Moderate stress	226 (25.9)	648 (74.1)		0.261 (0.131 – 0.520)*
High stress	27 (65.9)	14 (34.1)		1.000
**Caregiver’s burden**				
No – mild	14 (15.1)	79 (84.9)	12.600 (0.002)	0.872 (0.417 – 1.820)
Mild – moderate	199 (28.9)	489 (71.1)		1.390 (0.928 – 2.081)
Severe	47 (20.4)	183 (79.6)		1.000

Excluding perceived stress from the multivariable regression model, primary education (AOR = 1.62; 95% CI: 1.13 – 2.33), household income below ₦50,000 (AOR = 0.49; 95% C1: 0.25 – 0.96), and social support remained significantly associated with depression. The association between low social support and depression was somewhat stronger (AOR = 2.91; 95% CI: 1.40 – 6.06) in the model without perceived stress (S1 Table).

#### Factors associated with anxiety disorders among WCLHIV.

In Table 5, social support and perceived stress were significantly associated with anxiety disorders at bivariate level. However, at multivariate logistic regression, only perceived stress was a predictor of anxiety disorders among the respondents (AOR = 0.07; 95% CI: (0.02 – 0.22). The multivariable logistic regression model for anxiety showed no evidence of poor fit based on the Hosmer–Lemeshow test (χ^2^ = 5.11, df = 8, p = 0.746) and had a Nagelkerke R^2^ of 0.079.

**Table 5:** Factors associated with Anxiety among women caregivers living with HIV.

Variables	(n = 1011) Anxiety disorder No anxiety disorder N (%) N (%)		χ2(p-value on bivariate analysis)	AOR in multivariate analysis (95% CI)
**Age of the respondents (years)**				
18 – 49	139 (18.5)	611 (81.5)	0.805 (0.370)	
≥50	55 (21.1)	206 (78.9)		
**Marital Status**				
Married	104 (17.1)	505 (82.9)	4.437 (0.109)	0.816 (0.458 – 1.457)
Widowed	70 (22.6)	240 (77.4)		1.137 (0.632 – 2.045)
Single	20 (21.7)	72 (78.3)		1.000
**Educational attainment of the respondents**				
No formal education	9 (18.8)	39 (81.3)	0.057 (0.972)	
Primary	44 (19.7)	179 (80.3)		
Secondary and above	141 (19.1)	599 (80.9)		
**Employment status**				
Unemployed	5 (29.4)	12 (70.6)	1.165 (0.280)	
Employed	189 (19.0)	805 (81.0)		
**Employment status of the spouse**				
Unemployed	3 (21.4)	11 (78.6)	0.192 (0.717)	
Employed	101 (17.0)	494 (83.0)		
**Level of education of the spouse**				
No formal education	2 (8.3)	22 (91.7)	2.873 (0.238)	
Primary	23 (21.5)	84 (78.5)		
Secondary and above	79 (16.5)	399 (83.5)		
**Personal relationship with Health worker**				
Yes	51 (20.5)	198 (79.5)	0.356 (0.551)	
No	143 (18.8)	619 (81.2)		
				
**Number of living children**				
0	3 (27.3)	8 (72.7)	0.557 (0.757)	
1 – 2	92 (19.5)	380 (80.5)		
≥3	99 (18.8)	429 (81.3)		
**Currently providing care for a child living with HIV**				
Yes	19 (17.3)	91 (82.7)	0.292 (0.589)	
No	175 (19.4)	726 (80.6)		
				
**HIV status of the spouse**				
Positive	37 (15.2)	206 (84.8)	0.001 (0.982)	
Negative	41 (15.3)	227 (84.7)		
				
**Household size**				
<5	114 (21.0)	429 (79.0)	2.466 (0.116)	1.171 (0.834 – 1.645)
≥5	80 (17.1)	388 (82.9)		1.000
				
**Household income per month (Naira)**				
<50000	128 (19.2)	537 (80.8)	1.104 (0.576)	
50000 – 99000	60 (19.9)	241 (80.1)		
≥100000	6 (13.3)	39 (86.7)		
				
**Duration of living with HIV (months)**				
<120	79 (22.2)	277 (77.8)	3.194 (0.074)	1.886 (0.335 – 10.611)
≥120	115 (17.6)	540 (82.4)		1.000
				
**Duration on ART (months)**				
<120	80 (21.9)	285 (78.1)	2.743 (0.098)	0.615 (0.109 – 3.464)
≥120	114 (17.6)	532 (82.4)		1.000
				
**Viral load (copies/ml)**				
<1000	185 (18.9)	794 (81.1)	1.702 (0.192)	0.623 (0.276 – 1.405)
≥1000	9 (28.1)	23 (71.9)		1.000
				
**Presence of other co-morbidities**				
Yes	26 (17.8)	120 (82.2)	3.322 (0.190)	2.122 (0.961 – 4.687)
No	154 (18.8)	663 (81.2)		1.039 (0.639 – 1.692)
I don’t know	14 (29.2)	34 (70.8)		1.000
**Social support**				
Low support	12 (29.3)	29 (70.7)	6.332 (0.042)	1.587 (0.702 – 3.586)
Moderate	145 (20.2)	574 (79.8)		1.256 (0.816 – 1.933)
High	37 (14.7)	214 (85.3)		1.000
				
**Stigma**				
Low stigma	42 (19.8)	170 (80.2)	0.067 (0.796)	
High stigma	152 (19.0)	647 (81.0)		
				
**Stress**				
Low stress	4 (4.2)	92 (95.8)	33.544 (<0.001)	0.066 (0.020 – 0.217)*
Moderate stress	171 (19.6)	703 (80.4)		0.327 (0.169 – 0.632)*
High stress	19 (46.3)	22 (53.7)		1.000
				
**Caregiver’s burden**				
No – mild	10 (10.8)	83 (89.2)	5.270 (0.072)	0.505 (0.227 – 1.123)
Mild – moderate	134 (19.5)	554 (80.5)		0.794 (0.535 – 1.178)
Severe	50 (21.7)	180 (78.3)		1.000

Excluding perceived stress from the multivariable regression model, caregiver burden was significantly associated with anxiety. Women with no-to-mild caregiver burden had lower odds of anxiety compared with those with severe caregiver burden (AOR = 0.41; 95% CI: 0.19–0.90), (S2 Table).

### Qualitative findings

#### Participants’ profile.

The age of the participants in the FGDs ranged from 18 to 50 years, with a mean age of 38 years. They were 4 groups in all, consisting of 35 women caregivers living with HIV. They were 15 traders, 10 farmers, 6 civil servants, and 4 housewives. Most of them (60%) had attained secondary school education, and 70% of them had 2 – 3 children.

Five themes emerged from the focus group discussions as stressors of mental health. These include Stigma, pill burden, poor social support, fear of disclosure of status, and cost.

#### Stigma.

Most of the respondents reported that they have been stigmatized in one way or another by their family members, healthcare workers, neighbours, and in the workplace, which has led to their sadness and depression.

*“When I gave birth recently, my family got to find out and they were not happy. So, it caused problem between me and my family. Each time I remember it, I find it too difficult to eat, go out. Even my neighbours found out and tagged me “HIV people”. Whenever I remember all these things, it makes me unhappy”(Participant 2, FGD 1, 22 years old)*

*“Another thing is that, you should tell people to stop isolating us. They see us as people that will die soon.” Some of the doctors and nurses talk to us and treat us as though we are not human beings. It makes me depressed.” (Participant 9, FGD1, 40 years old)*

*“There are some jobs we apply for, such as nanny jobs, once they hear of our status, we get disqualified.” (Participant 2, FGD3, 46 years old)*

#### Pill burden/unavailability of drugs.

Some WCLHIV narrated their ordeal with taking the anti-retroviral drugs, which makes them anxious and causes fear of unknown.

*“The stress alone, you know I’m still young, ah for... I’ll say this thing now for the past twelve years I have been dealing with this drug thing and it’s not easy for me. I’m still young. How long will I go? That’s the thought I have been having, and it makes me worried” (participant 8, FGD1, 25 years)*

*“I always feel like my life will depend on taking drugs every day, that when will this thing, taking drugs end. That I don’t want to continue my life by taking drugs all the time” (Participant 6, FGD 2, 33 years)*

*“Most of us are not happy taking the drugs. If you are eating and remember you will take a drug, your heart will start beating faster,” (participant 3, FGD 3, 45 years)*

A participant had a different opinion on anti-retroviral medications. She narrated her fear of possible unavailability of the medication

*“Sometimes you think, with the way things are going, they might stop bringing these drugs into the country. What are we going to do? That means we will all die. So, it makes me scared and worried”. (Participant 4, FGD 4, 24 years)*

#### Poor social support.

Some of the participants reported experiencing poor social support characterized by domestic neglect, financial deprivation, and spousal discrimination which leads to depression and reduced capacity to care for their children.

*“….My children and husband are treating me badly because of my status and they don’t give me money to buy things and this makes me depressed and life is not being easy, so we are pleading with the government to help us …” (Participant 4, FGD 2, 50 years)*

*“…In our family, if your husband doesn’t have HIV, he isolates you and does not provide for you. And if your family is not financially stable, nobody can help you. When you remember how your husband treats you badly, all of these things can make you depressed. The things you are meant to do for your children, you can’t do them. You aren’t strong enough to fight for yourself, and for your children.” (Participant 6, FGD 3, 40 years)*

However, a woman caregiver had a different narrative

“My husband who was very supportive is late. The loneliness is too much. If my husband was still alive, he would have helped. Now, I do everything alone. Whenever I think of the fact that my husband is no more, I feel sad at times. (Participant 3, FGD 3, 45 years)

#### Fear of disclosure of status.

A few of the participants expressed fear of their HIV status being disclosed, leading to discretion in managing their treatment, and consequently contributing to feelings of anxiety and depression.

*“Anytime I remember that I need to go to the clinic and get my drugs, I get so uncomfortable and anxious. I think of how to handle the drug without anybody recognizing me. Even at my place of work, I don’t tell anyone where I am going and we are dying in silence.” (Participant 9, FGD 1, 40 years)*

*“I’m depressed because not everywhere I go to that I’ll take my drugs and it’s giving me stress” (Participant 6, FGD 2, 33 years)*

#### Cost of transportation and laboratory investigations.

Some participants highlighted the huge toll the costs of transportation to the clinic and some laboratory investigations takes on their finances and the impact these have on their mental health

*“I can’t meet up with appointment because of the nature of my job. The little salary I get, I spend it on lab test and transport. I am very disorganized.” (Participant 2, FGD 1, 22 years)*

*“I’ll say my going and coming to the clinic will cost about sixty thousand. Just to come to the clinic is thirty thousand, and I have to lodge. I lodge six thousand, and I’m still going back, so I get depressed whenever I remember how much I spend to get treatment.” (Participant 8, FGD1, 25 years)*

Integration of the quantitative and qualitative findings is presented in S3 Table, highlighting areas of convergence, complementarity, and divergence.

## Discussion

The prevalence of depression in this study was 25.7%, indicating that about one-quarter of the women caregivers were depressed. This rate is slightly lower than the 35.6% reported in Kenya among women living with HIV, but higher than the 5.3% reported in a Nigerian study [26,27]. These differences may be explained by variations in study populations and the psychometric properties of the screening tools used to assess depression [12]. In addition, 19.2% of the participants had anxiety disorders, which was comparable to the 20.8% reported in a global systematic review and the 21.1% observed in a study from Ethiopia [28,29]. While our study focused specifically on women caregivers, the other studies included women living with HIV more broadly. Nevertheless, our findings showed that women caregivers, just like other women living with HIV, are prone to anxiety disorders and depression. Also, some participants reported the combined demands of managing their own health while providing care and emotional support for their children as an additional source of psychological burden [30].

Furthermore, a substantial burden of psychological distress was observed among WCLHIV, with 79.0% experiencing high perceived stigma. As studies specifically on WCLHIV are limited, comparison with studies among women living with HIV showed a higher rate than the 69.7% reported in Iran, 66% in India, and 49.8% in Ethiopia [29,31,32]. This high level of stigma may be explained by the fact that, in many cultural settings, caregiving is associated with moral expectations of purity, strength, and responsibility. As a result, women caregivers living with HIV may be perceived as falling short of these ideals, thereby facing greater community scrutiny, judgment, and social blame [33]. Regarding other psychological characteristics, more than half of the respondents reported moderate levels of social support and stress, as well as mild to moderate caregiver burden. These findings may stem from their caregiving roles and were consistent with studies from Ethiopia, Nigeria, and India, which similarly reported moderate levels of social support, stress, and caregiver burden among women living with HIV, respectively [12,34,35]. This underscores the need for improved psychosocial interventions to strengthen social support networks, reduce stress, and alleviate caregiver burden among women caregivers living with HIV.

### Socio-demographic and clinical factors associated with depression

The high proportion of employed participants and spouses may reflect the broad definition of employment used in our study, which included informal and self-employed occupations such as trading, farming, casual work, and artisanal activities in addition to formal employment. Despite the high employment rate, most households reported relatively low monthly income, suggesting that employment did not necessarily indicate economic security. The predominance of economically active women may therefore limit the generalisability of the findings to unemployed women or those with greater socioeconomic vulnerability.

Participants with primary education were 1.5 times more likely to be depressed compared to those with secondary and above education. This finding is consistent with another study and implies that poor education may limit health literacy, economic opportunities, and coping abilities, thereby increasing vulnerability to depression among WCLHIV [12]. Surprisingly, our quantitative findings noted that participants who earned <50,000 naira had 58% lower odds of depression compared to those who earned above the income level, which contradicts our qualitative findings, as some of the participants narrated that they couldn’t afford to pay for the cost of transportation and laboratory investigations linked to their care, which leads to depression. The discrepancy between the quantitative and qualitative findings may stem from reporting bias and sample differences. Some participants may have underreported or misclassified their income in the survey, creating an apparent inverse link between income and depression. Moreover, qualitative participants were likely those facing greater financial and emotional strain, which may have amplified reports of economic hardship and its psychological impact. Nevertheless, several studies have reported that HIV positive women with low income levels are more likely to be depressed [26,30,36]. These findings highlight the importance of addressing financial barriers to healthcare and basic needs among WCLHIV. However, future studies should examine the relationship between household income and depression using broader measures of socioeconomic circumstances, including household wealth index, type of employment, and household expenditure, to determine whether these factors confound or modify the association between household income and depression. Longitudinal studies would also be useful in clarifying the temporal relationship between changes in economic circumstances and depressive symptoms.

### Psychological factors associated with depression

WCLHIV who reported low social support were twice more likely to experience depression compared to those with high social support. This finding aligns with our qualitative narratives showing that emotional neglect, isolation, and lack of financial help from family members lead to depression. Some of the participants felt mistreated by their spouses and helpless in supporting their children, underscoring how inadequate social support can hinder their caregiving role. As this may not only increase the women’s psychological vulnerability but may also negatively impact their children’s social and mental well-being [37]. Furthermore, respondents with low perceived stress had approximately 95% lower odds of depression compared with those with high perceived stress. This corroborates other studies done in Nigeria and China, illustrating that interventions targeting the reduction of perceived stress will alleviate depression in WCLHIV [18,38].

Although the quantitative findings indicated that stigma was not a significant predictor of depression, the qualitative data revealed that stigma remains a major contributor to depression among WCLHIV. Participants reported being stigmatized by family members, healthcare workers, neighbours, and employers, leading to feelings of sadness, isolation, and depression. Some recounted being labelled as “HIV people,” denied employment opportunities, or treated unfairly by health professionals. These experiences, supported by previous researchers, highlight how pervasive stigma within families and institutions continues to undermine the mental well-being of WCLHIV, despite efforts to promote acceptance and reduce discrimination [39,40]. This underscores the need for multi-level, context-specific interventions aimed at reducing HIV-related stigma within families, communities and healthcare settings.

Excluding perceived stress from the multivariable regression model, educational attainment, household income, and social support remained associated with depression. The association between low social support and depression became somewhat stronger, indicating that this association remained robust when perceived stress was excluded from the model. Also, the association between perceived stress and depression should be interpreted cautiously because of the conceptual overlap between perceived stress and depressive symptoms.

### Socio-demographic and clinical factors associated with anxiety

The qualitative findings revealed that pill burden affected the mental health of the WCLHIV. Participants expressed worry about taking the anti-retroviral medications for many years and being anxious each time they needed to take the medications. This was a similar experience of the PLHIV in eight high income countries who experienced emotional challenges due to daily medication [41]. It reflects the psychological burden of lifelong treatment and emphasizes the importance of integrating psychosocial support interventions to promote sustained adherence to antiretroviral therapy [42]. Nevertheless, a participant expressed her fears about possible unavailability of these drugs in the face of an unprecedented funding crisis and major cutbacks to HIV programme. This calls for collaborative efforts among governments, donors, and implementing partners to rethink sustainable financing strategies, ensure uninterrupted drug supply, and safeguard the health gains achieved over the years, which will ultimately improve the mental well-being of WCLHIV [43].

### Psychological factors associated with anxiety

Respondents with low perceived stress had 93% lower odds of experiencing anxiety compared with those with moderate-high perceived stress, which was consistent with the findings of other studies [18,34]. This implies that stress management is critical in maintaining good mental well-being and improving the overall quality of life of the women caregivers living with HIV, which in turn enhances their ability to provide effective care to their dependents [4].

Excluding perceived stress from the regression model, caregiver burden became significantly associated with anxiety. This suggests that the inclusion of perceived stress may have influenced the observed associations with other psychosocial factors. The strong association between perceived stress and anxiety should therefore be interpreted cautiously, given the conceptual overlap between perceived stress and anxiety symptoms.

### Psychological factors associated with anxiety and depression

From the qualitative perspective of our study, a key psychological factor contributing to anxiety and depression among WCLHIV was the fear of disclosing their HIV status. Participants described experiencing deep emotional distress stemming from the ongoing effort to conceal their status and the constant vigilance required to ensure that others did not discover their use of antiretroviral therapy. These coping strategies adopted to maintain secrecy have, in turn, resulted in heightened anxiety and depressive symptoms. This may be attributable to fear of stigma and discrimination as evidence has shown that people living with HIV still face social judgement on disclosure of their status leading to poor adherence to medication and retention to care [44].

A key strength of this study is that, it is one of the few studies to examine mental health disorders specifically among women caregivers living with HIV including a large sample size. The mixed-methods approach enriched the findings by capturing both the quantitative burden and the contextual experiences underlying these mental health disorders in WCLHIV. Nevertheless, the sample size was originally estimated using the prevalence of comorbid anxiety and depression from a comparable population; as depression and anxiety were analysed separately, using separate prevalence estimates for each outcome and selecting the larger required sample would have been a more conservative approach. Women receiving treatment for a mental disorder and those too physically unwell to participate were excluded, which may have led to underestimation of depression and anxiety and reduced representation of women with greater health needs. Future studies should consider deferred recruitment of temporarily unwell participants to improve representativeness. The cross-sectional study design limits the ability to establish causal relationships between the identified factors and mental health outcomes. Social desirability bias cannot be entirely excluded, as the questionnaire was interviewer-administered and some data collectors were healthcare workers in the study clinics. Participants may have been reluctant to disclose psychological distress because of concerns about how such disclosures might be perceived by clinic staff or whether they could affect the care they received. This may have resulted in under-reporting of some psychosocial problems and consequently underestimated their prevalence. However, this was minimized by emphasizing the importance of honest responses and assuring participants of the anonymity and confidentiality of their information, which was used solely for research purposes.

## Conclusion and recommendations

This study highlights a high burden of depression and anxiety among women caregivers living with HIV, characterized by high levels of perceived stigma, moderate social support, and moderate stress. Low social support and stigma emerged as key contributors to depression, while high levels of stress and pill burden were strongly linked to anxiety. Moreover, the fear of disclosing one’s HIV status was a central psychological factor underlying both anxiety and depression among WCLHIV. Targeted multi-level psychosocial interventions and community empowerment programs should be implemented to strengthen social support systems, reduce perceived stigma, and build coping skills for stress management among women caregivers living with HIV. Effective counseling approaches like motivational interviewing and cognitive-behavioral techniques should be deployed to address disclosure fears and adherence-related stress, such as pill burden to improve psychological well-being.

## Supporting information

S1 Data Supporting dataset used for the analysis presented in this study. (XLSX) (XLS)

S1 Text Supporting dataset used for the analysis presented in this study. (DOCX)

S1 Table Multivariable logistic regression analysis for depression excluding perceived stress. (DOCX)

S2 Table Multivariable logistic regression analysis for Anxiety disorder excluding perceived stress. (DOCX)

S3 Table Mixed-methods mapping of quantitative and qualitative findings. (DOCX)

S1 Checklist STROBE checklist. (DOCX)

S2 Checklist COREQ checklist. (DOCX)
