# Supplementary material for: “Carrying the burden silently”: A mixed-methods analysis of common mental health disorders among women caregivers living with HIV in Enugu, Nigeria
Source: PLOS Ment Health. 2026 Oct 23;3(10):e0000764. doi: 10.1371/journal.pmen.0000764 (PMC13693887; doi:10.1371/journal.pmen.0000764)
Supplement: Supplementary file 1 [file pmen.0000764.s002.docx]

**Transcript of FGD 1 with women caregivers living with HIV**

| **INTERVIEWER** | **RESPONDENT** |
| --- | --- |
| **The first question I want to ask you is that since you became aware of your HIV status, how did it affect your mental health well-being? From the very first time you knew; how did you feel?)** | R8: for me I get angrily easily, I get angry easily and I’m depressed because not everywhere I go to I’ll take my drugs and it’s giving me stress because of my work and everything just stress. I can just get pissed off, like this morning when you called, I was so angry because I have to say not less than... I’ll say my going and coming to the clinic will cost about sixty thousand. Just to come to the clinic is thirty thousand, and I have to lodge. I lodge six thousand, and I’m still going back, so I get depressed whenever I remember how much I spend to get treatment.” |
| **Yea thank you (Thank you. Is there any other person? Number two wants to talk)** | R2: I am number two. Thank you for this organization. I am really happy. Because for me (this really affected me so much. When I got HIV, I felt like dying but to God be the glory I got myself back. I didn’t get married to the person I was engaged to but I eventually got married and gave birth to children. My challenge is that I don’t go to places I want to go, I can’t eat what I want to eat like coconut, as long I’m taking my drugs I can’t eat it. I can’t meet up with appointment because of the nature of my job. The little salary I get, I spend it on lab test and transport. I am very disorganized.” |
| **Thank you. (Is there any other person? Number five)** | R5: (I didn’t know when it started. It started as a stomach pain and running stomach. I drank all kinds of medicine. I went to park lane and I was referred here to UNTH where I was given medicine) |
| **(How did it affect your mental health?)** | R5: (I couldn’t eat anything until the money and drugs I was given before I was able to eat) |
| **(Mummy please try to speak up. Let’s ask number one. Let number one contribute)** | R1: (Please I thank the people that organized the meeting. Nobody is truly happy when they are ill. The condition of the country isn’t helping either, my children and husband are treating me badly and giving me headache and life is not being easy, so we are pleading with the government to help us to reduce the stress. I greet you all) |
| **Number 4... Number 4** | R 4: (I greet everyone. The first I got the infection was when I gave birth recently. My husband first contracted it and didn’t tell me. What you people should do is that, when you ask people to come and collect drugs, if they are married then their spouses have to be present when they’re collecting their drugs. My husband has been collecting his drugs for years when I hadn’t contracted it. I gave birth to my first child who is infected now. It was when I gave birth to my second child that I found out my status, luckily, he was just four months old. I took my first child to the hospital for test and found out, he was positive. I began to ask questions, that’s when he brought me here. My second child didn’t contract HIV. The reverend took care of my child well. Each time I remember the incidence, I get angry. |
| **(Thank you. Number nine)** | R9: Number 9. I want to thank the people that organized this meeting. God bless you all, it’s not easy. When I first found out about my status, it felt like my world was over, but I was able to pull myself together. Anytime I remember that I need to take my drugs or I need to travel, I get so uncomfortable. I think of how to handle the drug without anybody recognizing me. Even at my place of work, I don’t tell anyone where I am going and we are dying in silence. If there is a way you people can do it.... most of us are not happy taking the drugs. So, I thank you all, if there’s a way you people can help us, please do. |
| **(Thank you. Number seven)** | R7: Number seven. I greet everyone. When this thing started. I thought I was going to die but I thank God for today. Anytime I come to collect drugs, the doctors always encourage me but I want to plead with you, the price of the lab test, 5500, it is difficult for some of us. Please find a way to help us. I thank God for the doctors and nurses that attend to us, they give us helping hands. |
| **(There’s no problem. Thank you. Is there anyone that want to talk.**  **Okay than you (Thank you. Let’s move forward) Based on experience (what is experience? You people know what is experience? Based on your experience, what are the things, factors that affect our mental health?** | R2: Number two (our experience... whenever we remember that we are taking our drugs, and we have HIV, we get angry. For example, when I gave birth recently, my family got to find out and they were not happy and I also delivered through CS. So, it caused problem between me and my family. Each time I remember it, I find it too difficult to eat, go out, even my neighbors found out and tagged me HIV people. Whenever I remember all these things, it makes me unhappy) |
| **Number one** | R1: (Another thing is that if your husband got it first and hid it from you and you found out later. Whenever you see your husband, you feel like hitting him but you calm down when you see people around, you begin to have headache) |
| **(Thank you. Number six)** | R6: (My experience is from some of your doctors and nurses). I could remember that when I so first of all discovered it in me. Instead of to hide it and guide me, she went and told her siblings. They now made it known to people. When I’m passing, they will say you have HIV, each time I see that nurse I get upset. That is all. |
| **(Thank you) Number eight** | (silence) |
| **(Pause it)** | R8: Each time I remembered my ex that gave me this thing, I just hate men for no reason. Like I don’t have boyfriend and... I don’t trust people because of that, I don’t trust. I lost trust on people because of it. The stress alone, you know I’m still young, ah for... I’ll say this thing now for the past twelve years I have been dealing with this and it’s not easy for me. I’m still young. How long will I go? That’s the thought I have been having. |
| **Thank you. (Thank you. Is there any other person. Is there anyone here experiencing family issues coupled with your status... Does family factors that contribute to your mental health?)** | All: Yes  R1: Number one |
| **Number one** | R1: If you want to run test in the lab, lab fee is five thousand five hundred naira. You will buy what your family will eat. Instead of running for test, you use the money to eat and be happy |
| **(Thank you. Is there any other person? Number nine do you want to talk?)** | R9: when you remember ... just like when you come. They will tell you that it’s time to run the test and you don’t have money. You will become confused and disorganized. |
| **(Thank you. Number two)** | R2: (This is very important; it’s concerning the lab issue. When you go to talk to people in the lab)  The way they will talk to you, you might... that is slumped immediately...  (madam please go if you don’t have money now, you can come when you have the money). They will not even.... that’s... pet you, you know this our condition needs petting. The way they will talk to you; you will say let you go and hang yourself immediately. Please you people should do something about it |
| **(Thank you. Is there other person that has anything to say about family issues contributing to this? Anyone?)** | No response |
| **Okay. Thank you.**  **(Since you all responded no, I want to know, what do you think in your opinion, is there anything you think we can put in place or do to improve mental health amongst people with HIV. I don’t know if you people understand me? Is there structure you think we can put up here to make the mental health better. I don’t know if you understand. Do you understand?)** | R7: Laughs, You can look into the lab fee, five thousand five. It will help our mental health to improve |
| **Number nine** | R9: (Another thing is that, you should tell people to stop isolating us. They see us as people that will die soon) |
| **(who are those that are isolating you?)** | R9: (Ehh, those that feel that they are not infected with the sickness, in this place) |
|  | R9: (Here, here. Some of the doctors and nurses talk to us and treat us as though we are not human beings. It makes me depressed. |
| **Number two. Thank you so much.** | R2: (I want to thank the people that organized this meeting, this meeting made me happy. What I want to say is that, you people should try and get a department here and get kindhearted people to attend to us. For example, during the covid 19 period, there was a day I forgot my nose mask. The nurses shouted and spoke to me in a demeaning manner. Please it will be better for all of us if mental health became a department here) |
| **(Thank you) Number one** | R1: (Number one. Just like what number nine said, a solution is needed. Most of the doctors and nurses avoid us. Some of us got HIV while giving birth or taking injection. It’s not only contracted through sex. Two, that’s why I am not in any support group. There is something that government gives.... Old people came from far to take the drugs and run test. The leaders of the support group embezzled the drugs and when the older people came, they heard that the drugs had finished. If only they will help as they should, they should also contribute with food and little token)  (everyone claps) |

**Transcript of FGD 2 with women caregivers living with HIV**

| **INTERVIEWER** | **RESPONDENT** |
| --- | --- |
| **(So, I can record right). So, the first question is eh (since we got to know of our status and started coming here to collect drugs, how has it affected our mental health) since we started taking... since we were told our status in this hospital, how has it affected your mental health? Did you get it?** | R1: Okay |
| **How has it affected your mental health?** | R1: Okay since I noticed my status and ehh since I started taking my medication, I noticed that whenever I took the drug, my brain will be... it’s affecting my brain. I will be reasoning abnormally. So, that’s the way it’s affecting me |
| **How does it... ehm I just want to know, how do you reason... what do you mean by saying you reason abnormally?** | R1: That’s uhmm once I take the drugs, my head will be like, is it compacting or what? I don’t know the English I’ll use. It will be doing me somehow. Then I complained to the doctor and he said the medicine is a hard drug, that’s why it’s doing me like that. That I have to change my drug, that I have to change the medicine. Which they have already changed for me |
| **So ehh apart from the drug that you’re taking hmm,**  **How has your mental health been since you knew that, you are positive?** | R1: Okay? At times I get angry easily, maybe a little thing I will get upset. You understand? |
| **Okay?** | R1: That’s what I noticed |
| **So, who else has something else to tell us (anybody else)? Who else has something else to tell us. You know why I’m speaking Igbo... English, because one of us is not Igbo. But if you still want to talk to us using Igbo, please you are free.** | Silence |
| **Number? Number 6. Okay number 6.** | No response |
| **(So does anyone have any question or contribution). I asked since you knew about your status, how has it affected mentally, emotionally, how has it affected you? See eh, this thing is not... we need your (interrupted)** | R2: It’s not affecting me anything, because it’s only first time wey I take that drug, I feel somehow that day. I reach like that three days I cannot get up. It’s only the first time |
| **I’m not really asking about the drug, what the drug is doing to you. I’m asking your own, when you knew that your status now, you are positive. How has it affected you since you knew it... from the first time you knew your status?** | R2: Only the first time |
| **OK. What did it do it you? That’s what I want to know** | R2: I vex |
| **That what?** | R2: I vex that time, but all everything, doctor later... I’m not happy that time. Doctor call me back, talk to me. Talk normal thing for me before I get myself |
| **Thank you. Who else? Number 6** | R6: I was like my life is ending  R6: Yes. I was like my life is ending. I was like my life is no more... there’s nothing, no value again |
| **Okay?** | R6: I was not happy |
| **But now, how are you feeling?** | R6: That’s I’m still feeling like that, but not much |
| **Okay, okay. Who else has something to say? Number two, do you have anything to say?** | R2: No, no |
| **Okay. So, then I want to ask? (inaudible speech) So, I want to ask eh, apart from these things that we have discussed, is there another person that want to share something with us? Since you knew about your status, since you knew about your status, how has it affected you? Apart from being angry and the vex, is there another thing it does to you? Because these are the things we need to know.** | R6: I don’t want anybody to notice that I have such a like that. Because anytime, any day I don’t people to notice... |
| **You don’t want people to know what?** | R6: My status |
| **Okay, so it makes you to hide or what?** | R6: To hide, to feel somehow |
| **To feel somehow. What do you mean by feeling somehow?** | R6: Like people knowing my status.  I’m depressed because not everywhere I go to that I’ll take my drugs and it’s giving me stress’’ |
| **So, what else, what else (is there no other person?) So, are you saying that since you know your status, apart from these ones you’ve said, there’s nothing else that you’ve experienced?** | R1: Another thing is that... (Interviewer cuts in) |
| **Number 1** | R1: Okay I’m number one. Once I stopped taking my medication, I stop... I start emaciating. And ehh, you know some people used to hide, even to come and take their drugs they will be hiding. Me I doesn’t hide because I don’t see as a bad thing. It’s a normal thing that can happen to anyone. You understand. So, I used to come around to take my drugs, my medication and that’s it. |
| **So, okay thank you. So, there’s nobody else? (there’s no other person that has...) It mustn’t be English like I said (If you don’t know how to speak English) you can put it in Igbo, we will all translate it (you understand). The most important is let’s discuss cause (this discussion is very important to us, so there won’t be any complaint from anyone). In short, I have discussed this with other people. So, number 2 (please what do you have to tell us) since you were told about your status. The first you were told, you didn’t feel anything, even till now?** | R2: No, I didn’t feel anything |
| **You are, okay?** | R2: yes |
| **Number 4, okay** | R4: (I didn’t feel anything. Just that sometimes I feel guilty) |
| **Okay** | R4: So that’s it. I don’t feel anything |
| **Sometimes you feel guilty. Why do you feel guilty? (what makes you feel guilty)** | R4: Because this kin thing as in (I wasn’t expecting it) eh ehn. So, and again sometimes (I feel like my husband is always angry with me so I was like if I had known I wouldn’t) |
| **Marry him?** | R4: Yes. “….My children and husband are treating me badly because of my status and they don’t give me money to buy things and this makes me depressed and life is not being easy, so we are pleading with the government to help us |
| **Okay, alright. Thank you. (please does anyone have anything to say?)** | R6: what I have to say is that.... (interviewer cuts in) |
| **Number what?** | R6: Number 6.... is that, I always feel like my life will depend on taking drugs every day, that when will this thing, taking drugs end. That I don’t want to continue my life by taking drugs all the time |
| **Okay** | R6: So that’s how I always feel |
| **Okay you always feel?** | R6: When will I end taking drugs |
| **Okay** | R5: I’m number five |
| **Number five, okay.** | R5: The first time I heard about my status. I was not happy. After while, I saw a doctor and he gave me some advice. Since then, I was calm and taking my drugs normally |
| **But since then, you were calm. Sometime do you feel that emotional (respondent interrupts)** | R5: I don’t feel it because I know it has happened; it has happened so there’s nothing I can do. |
| **Alright. Thank you, okay. Do you want to say something?** | R: No, I don’t have anything (faint voice) |
| **Based on our experience now, hmm, based on our experiences now, I want to know what are the things, what are the factors? (Pause)**  **So, I want to ask, this is our experience. It may not be your own, it may be that you have been discussing with your fellow women. We want to know, what are the things that you feel, factors, what are the problem, what are the issues that can affect our mental health. Did you get?** | R1: A divorce can |
| **Divorce?** | R1: Yes, can affect someone’s mental issue |
| **Divorce (How?) how?** | R1: Marriage. When you are into marriage. Divorce can affect mental issue. When you are thinking what you don’t supposed to think, it can your mental issue as well. |
| **Okay. Apart from that, what else do you feel as young mothers, are the things that can .... we are living in Nigeria, we are you know, everything, not about drugs. We are looking at it from our health facility, we are looking at it from our family. Do you get what I’m trying to say?** | No response |
| **We are looking at it from everything. What are the things that you feel you are facing, or your friends that are taking these drugs, that are also your agemate, that you interact with. Based on their experience that you found out that these are the things that are affecting their emotional health or mental health? Do you get?** | (background noise) |
| **Number?** | R6: Number six. When there is no money to take care of yourself enough. You are (interrupted) |
| **When there’s no money for you to do what?** | R6: To take care of yourself |
| **Okay?** | R6: And feed the family |
| **Thank you** | R6: It affects mentally |
| **Okay. Thank you, thank you (Please any other person?). Who else? You wanted to say something?** | R1: I have said it already |
| **Please, we are all in the country, we are all here. We are not asking about... I’m just asking generally. What are the things? Everybody goes through mental health issues, one day or the other. It’s not that it’s meant for some people. So, I’m asking this generally.** | R1: Another thing, I’m number one |
| **Number one** | R1: Number... okay, number one. Another thing that can affect our mental health issue is lack of communication. When you don’t communicate with your loved ones, maybe you feel lonely. It can as well affect the... the health |
| **Okay** | R1: Yea, the mental health |
| **Thank you, thank you. Number one. So, in our heath facility, is there anything that, when you come here, that will affect your mental health? Let’s start from health facility. Is there any health facility thing you know in our clinic? Hmm? Number?** | R6: I’m number 6. Like as we came now and our folder have not been found since when we came. So, it affects us mentally |
| **Thank you. Number six, thank you. Who else?** | R1: You mean in this clinic? |
| **Yes, is there anything that affects your mental health?** | R1: Okay. Like here as we are now, there’s no light. Somebody like me, I feel disturbed when I stay in a place where there’s no fan or AC (background noise) Yes. I feel disturbed |
| **Okay, okay. Number 2. (There’s something) that you know, once this thing happens, you are not yourself again. You just feel no... no... no... this thing has really affected... this has really affected my mental health. It makes you worried, it makes you sad, it makes you something, do you get?** | (No response) |
| **What are the things? These are way to... you know, so we know and help.** | R2: I don’t want to talk anything |
| **(Even the child you are taking care of), is there not anything that you know that, (that can affect your mental health)? Hmm? (you don’t have?)** | R2: (No, I don’t have) |
| **What of you, number 3?** | R3: Number three. Now I’m feeling bad. The first-time wey I start for here, they tell me to do antenatal, do everything. I do everything. Later on, they come tell me say it’s not by force to do antenatal here, you can do antenatal for anywhere wey you live. So later the first one, the second one, I no come do antenatal here, I come do antenatal for another place. The day I wan come collect this viral medicine wey I will give my baby, they give me. But this one now, last time I suppose to collect that thing, they say no, say till I go where, register where, register where. I don’t know... I don’t know |
| **Okay, so the stress of you (background noise) of you getting drugs (respondents interrupts)** | R3: I suppose to collect that thing. I no suppose to enter motor and come here again but they still make me enter motor and come here again up to still |
| **Still, they’ve not given you. Thank you, thank you so much.** |  |
| **Do you have anything to say?** | R4: Hmm nothing |
| **Even if it is family, anything?** | R4: It’s just the stress is much |
| **Stress in terms of where now?** | R4: In terms of file. They will ask you go there, go this place. Before they will now take you to doctor |
| **Okay, thank you, number four** | R4: Ehh number four |
| **(Do you have any family issues, outside this health facility. We have heard of health facility now). I’m now asking outside here, is there anything outside that affects your mental health, that you want to share with us and you feel we can help you from there. Because the idea is to share. Did you get what I asked? Is there any mental health issue... family issue that you feel you can share with us?** | R6: No, I don’t have anyone |
| **Hmm?** | R6: I don’t have any  R6: Like at times when my mother will start talking about my status |
| **Number what?** | R6: Number six |
| **Okay six. Thank you** | R6: Talking about my status |
| **Your who?** | R6: my status... my mother |
| **Okay, okay alright** | R6: It affects me |
| **So, it affects your mental health? Alright thank you. So, let’s go.... so, what else now? Are we...? Is there anybody that has anything... anything to say in this number? In this question?** | (muttering sounds) |
| **Thank you. I think we have come to the end of this. Does anybody have any problem, any comment, any question to ask us?** | (silence) |
| **To ask me** | R1: I need to ask you question? My question is this, someone that have this issue? Is there any diet that the person will be placed on? Or can the person be eating anything? |
| **Diet?** | R1: Like food |
| **Like what do you mean by if someone has the issue? Which issue? Is it? Let’s understand** | R1: This our status now |
| **Okay this your status** | R1: Yes. So, is there any diet or food that the person will be placed on? That will be helping to... is it to neutralize the issue? |
| **Okay, thank you. I will answer it. I’m coming. (Does anybody else have any question?) No. Okay, thank you so much.** |  |

**Transcript of FGD 3 with women caregivers living with HIV**

| **INTERVIEWER** | **RESPONDENTS** |
| --- | --- |
| **(I want ask you. Since you got know of your status, how does it affect mental health? Since you got to know, how does it affect you mentally? I don’t know if you understand what I asked you?)** | All: No |
| **(You didn’t understand? I will repeat the question. Since you knew about your status, and you started collecting drugs from here, how does it affect you mentally? Have you understood now?) Should I ask it again?** | (Inaudible response) |
| **(Inaudible speech)** | (silence) |
| **(Do you want to say something mummy). Number nine. Go ahead** | R9: (I have been coming here since 1991. It’s not been easy on me mentally. I thank God that I started coming here, if anyone tells me to leave park lane I won’t do it. Since I started taking my drugs, I have been doing okay. Nothing is really wrong with me. The reason I came here is that I was told to come and speak up. I like what is going on here. Well done) |
| **(I heard you, but I’m not asking about this meeting is doing for you. What I’m asking is that since you started coming here, how has your mental health been? What I am asking is since you started collecting drugs, how have you been mentally?** | R9: (Is it whether I feel sick or if I’m feeling alright?)  (background noise) |
| **(We mean within you; how do you feel?)** | R9: I still don’t understand |
| **Number?** | R3: Number three. When I got to know of my status, I felt very angry. Before now, there are some kinds of job I am able to do that are strength consuming, but since I started taking these drugs, I get tired easily. Now, I am not able to do those kinds of jobs and they are the ones that pay more money. Also, I am not able to go to places I want to go because my last son is not old enough to take care of himself. I can’t be open in places about my status for fear that people might laugh at me, or run away from me. If I can be free of this illness, I will be able to open up to people, achieve a lot of things. That’s what disturbs me. Thirdly, my husband who was very supportive is late. The loneliness is too much. If my husband was still alive, he would have helped. Now, I do everything alone. Whenever I think of the fact that I am not feeling well or I am taking drugs or my husband is no more, I feel sad at times. The drug helps but there are sometimes I feel overwhelmed. That’s all I have to say) |
| **Thank you, thank you.**  **I2: (Does anyone have anything to say concerning the question we just asked)** | (no response) |
| **(Anyone? You know how it affects you. Like we said, this is a personal experience. There are no right or wrong answers. We explained this to you when we started. Please feel free to speak to us)**  **I2: (Anyone that what’s to speak in English should please speak, if you can speak in Igbo, it’s still okay. If you want to speak in both... Interviewer one cuts in)**  **I: (Everything)**  **I2: (Fine, we understand everything)** | (silence) |
| **(Does anyone want to talk)** | R1: (What I observed since I started taking this drug is that, I feel weak in my body all the time. I ask myself if it is the drug that is causing it. I started taking this drug since 2008. When I look at myself, I conclude that it is the drug that causes the weakness. This is something I haven’t experience before) |
| **(Thank you. Does anyone have anything to say? Does anyone have anything to say? Nobody?** | (No response) |
| **Mummy do you have anything to tell us? Tell us. Number what are you?)** | R5: I am number five |
| **Number five** | R5: I have known my status for a long time. I got really angry. I felt like my life was over. Everyone, including my children were wondering why I was always angry.  I thank God. It was when I started coming here to take my drugs that I started seeing people like me, I started going out. Then I calmed down, started doing things as instructed. My husband and children have been very supportive. That was my experience. I got rid of all that anger and issues I was having and became very active) |
| **(Thank you, thank you. Does anyone have anything to say. Number seven)** | R7: When it started, I didn’t know. I kept falling ill. In my place of work, a doctor asked me what was wrong with me and I told him I didn’t know. Finally, I got to know. The doctor there told me that this illness doesn’t kill anybody but that I should take my drugs as instructed. Since I started taking these drugs, it doesn’t affect that much anymore |
| **(Thank you. Let’s go to the next question. We are almost done. Please I want to plead with you especially those who have not said anything. I would like us to contribute to this discussion. This information will help us)** |  |
| **Based on your experience (background noise) (Based on your experience, what do you think are the things that contribute to your mental health issue? I don’t know if you understand my question)** | R: Yes |
| **(What are your thoughts? The way you understand it. What do you think can contribute.... It may not be you. It may be your friend, or anybody. We want to know what contributes to your mental issues in this facility. Did you hear me?** | R1: Yes |
| **Number one** | R1: Number one. From what I understand, what causes it, is when things aren’t going the way they’re supposed to. You are not happy and you don’t have peace of mind. You become very disturbed mentally. But if you are mentally okay, you can do things on your own) |
| **Thank you, thank you. There’s someone that raised her hand)** | R4: Number four |
| **Number four** | R4: What can affect someone mentally is overthinking. The problem we go through can affect someone mentally |
| **(Thank you. Number three. You want to say something)** | R3: When it comes to mental health problem, just like what my sister said, when things are not going well for you, When I think of the drug, I take all the time, it makes my heart beat fast and it gets me angry. Most of us are not happy taking the drugs. If you are eating and remember you will take a drug, your heart will start beating faster  When things are not going the way, they are meant to and you are not physically fit to do thing you want to do, it makes someone very upset. |
| **(Thank you. Number two, is there anything you want to tell us?)** | R2: (What I want to say is that), in a situation whereby you are looking for a job, you reach there and they ask you for your status and all those tests and it happens that you come out positive. It happens that they will not take you because, they find it difficult to take people on such status. |
| **What type of job?** | R2: There are some jobs we apply for, such as nanny jobs, once they hear of our status, we get disqualified’’ |
| **Thank you. Thank you. Is there anyone that wants to say anything? Number six** | R6: Number six. (What I understand is that, for example in our family, if your husband doesn’t have HIV, he isolates you and does not provide for you. And if your family is not financially stable, nobody can help you. When you remember how your husband treats you badly, all of these things can make you depressed. The things you are meant to do for your children, you can’t do them. You aren’t strong enough to fight for yourself, and for your children. Then you remember your condition, your husband, the condition of your children. It can affect you mentally and make you depressed |
| **Thank you, thank you. Does anybody else have anything to say? So, we can move to the next question** | (silence) |
| **Does anybody else have anything to say? Number eight, do you want to say something?** | (No response) |
| **Number ten, do you want to say something** | (silence) |
| **(Does anyone have anything to say? We are rounding up)** | (silence) |
| **(Nobody? Okay. Thank you everyone.)** |  |

**Transcript of FGD 4 with women caregivers living with HIV**

| **INTERVIEWER** | **RESPONDENTS** |
| --- | --- |
| **(Since you got to know about your status, how has it affected your mental health wellbeing? I don’t know if you understand what I’m asking you. Since you got you know that you are HIV positive, how did it affect your mental health well-being? Did you understand?)** | (Inaudible response)  R5: Actually, at first it was a real depressing emotional something. So, but with time I got to realize I have to live for myself cos my mum wants me to live. I have to live cos I have a child to take care of. Then at that period, doctors were able to talk to me, telling me that as long as I am taking my drugs, I can be whatever I want to be. So that’s how it started and that’s how I have been going since. |
| **Okay. So how has ... how did it... you said that when you started it affected your emotional... (respondent cuts in)** | R5: Yes |
| **So, what were the things that you noticed then?** | R5: I keep asking myself. Sometime I seat on myself, I was asking myself, how do I...? how did this thing happen? How do I get to...? like how did I find myself in this situation? Sometimes I lock myself in the house like three days, I am not out. My mum keeps getting worried and was like telling me “It’s not your fault”, because then we got to find out that my parents doesn’t even have it. Then, when we got to find out what... I found out when I was fifteen years. Then, my mum had to realize that it was the blood transfusion I had when I was five years. Because, it really affected me in a point that I have to start vomiting out blood, stooling blood, urinating blood. So, that was the kind of depressing action that this thing got to have me on. So, my mum told me that it’s not your fault, you don’t have to blame yourself, you don’t have to blame anybody that this actually happened. So, words of encouragement from my mum and from the doctors, I get to find myself out of the depressing character I found myself in |
| **I: Thank you. (Does anyone have anything to say? We can speak Igbo) Number two** | R2: Number one |
| **Number two (laughs)** | R2: (I am number two, okay. Since I started drinking this drug, it didn’t affect me so much, I do things that I want to do, I go to anywhere I want to go, as far I take my drugs.) |
| **(Okay. I am not asking about the drugs, what I am asking is, when you got to know you were HIV positive, how did it affect it you? How did you feel mentally?)** | R2: (I didn’t feel bad so to speak because I have heard about the drugs and if you take the drugs, you will be able to live longer. So, I began to drink my drugs.) |
| **Thank you. Number one** | R1: Number one. When I first find out that I was positive, it wasn’t easy. I thought of killing myself. It got to a point; my HIV status was all I was thinking about. I found out about my status when I was pregnant. It was so bad that I lost the pregnancy. It affected me badly, I won’t lie. It wasn’t easy. Whenever I remember those times, I get so upset. I haven’t been able to recover till now. Though, I have been taking the drugs for a long time now. |
| **I2: Like how many years?** | R1: about six to seven years now. (It’s not been easy for me. Whenever I remember I take drugs every day, I get sad. It’s not easy. Thank you) |
| **Thank you. (Does anyone want to say something)**  **I2: Number six** | R6: When I found out, I cried so much. I went through a lot then. I didn’t expect that something like this would happen to me. I didn’t recover even after a month. My relationship didn’t work out. I get panic attacks up till now. I suffered low BP two months ago and I nearly died. I thank God that I am alive today. |
| **Thank you. Number three** | R3: I have been taking this drug for eight years. It started out as headache, then fever. When I got to the hospital, I was told that, I was positive. I felt like dying, but when I saw other people like me, I became strong. Now, it doesn’t affect me that much again. I have given birth to three children safely.) |
| **(Okay thank you. Does anyone have something else to say? Another question I want to ask, based on our experience, what do you think contributes to the mental health problem among women)** | R2: (What can cause it is....) (second interviewer cuts in) |
| **I2: Number two** | R2: (Number two... is if the person thinks too much about her condition. That can cause it. But if the person accepts the situation and focuses on her health and take her drug, it won’t worsen it.) |
| **Number four** | R4: (Number four. Overthinking can cause it, when you don’t have what to eat. If you are able to eat and take your drugs you will be feel strong and healthy. When someone thinks about all these things, it can contribute to one’s mental issue) |
| **(Thank you. Mummy do you want to say something? We are asking what can worsen the mental health problem)** | (silence) |
| **(Mummy what you have anything?)** | (I don’t have) |
| **(We are all women. What we are asking... (second interviewer cuts in)**  **I2: (There’s no right or wrong answer)**  **I: (Yes, we are asking about the issues you encounter. For example, coming here to take your drugs, you have children, the economic situation in this country. What can you say affects your mental health. We need to hear the experiences you’ve had)** | R1: (I am number one. What I think contribute to mental issues is stress. Stress can bring someone down even someone that isn’t HIV positive. Stress can kill someone. Only if we can reduce the stress. The condition in the country isn’t good but we will still our best as human beings. But we need to reduce stress and also, overthinking can cause it. Once you start thinking a lot, it can lead to stress and it is not good for the body. Your body doesn’t need it for now until... in fact there’s nothing like until, your system doesn’t need stress or overthinking. When it comes to family issues, what you can do is concentrate on yourself. I feel like if we stop thinking about these things, it can reduce stress level and everything will be fine) |
| **(Thank you. Based on the fact that we are taking drugs, does it affect our mental health? As a woman taking these drugs, is there anything you think can damage our mental health?)** | R1: (What I think can damage our mental health is exactly what I said now, overthinking and stress.) |
| **(Okay thank you. Does someone want to say something? Number?)** | R2: (Number two. Number two. Another strong point is the situation of the country we live in now. It is serious. If we can get financial support, it will help a lot and make things a bit easy. That’s one of things that affects our mental health) |
| **Okay, thank you** | R4: Number four. Another thing that can worsen it, is the condition of the country. “Sometimes you think, with the way things are going, they might stop bringing these drugs into the country. What are we going to do? That means we will all die. So, it makes me scared and worried. |
| **Does anyone have anything to say or want to ask question?** | (unintelligible words) |
| **No question? Does anyone have any question? No question** | (No response) |
| **I thank you very much for your time.** |  |
