# Supplementary material for: “Carrying the burden silently”: A mixed-methods analysis of common mental health disorders among women caregivers living with HIV in Enugu, Nigeria
Source: PLOS Ment Health. 2026 Oct 23;3(10):e0000764. doi: 10.1371/journal.pmen.0000764 (PMC13693887; doi:10.1371/journal.pmen.0000764)
Supplement: Supplementary file 2 [file pmen.0000764.s003.docx]

**S1 Table. Multivariable logistic regression analysis for depression excluding perceived stress.**

| **Variables** | **(n=1011)**  **Depressed Not Depressed**  **N (%) N (%)** | | **χ2(p-value on**  **bivariate**  **analysis)** | **AOR in multivariate analysis (95% CI)** |
| --- | --- | --- | --- | --- |
| **Age of the respondents (years)** |  |  |  |  |
| 18 – 49 | 181 (24.1) | 569 (75.9) | 3.815 (0.051) | 0.941 (0.662 – 1.340) |
| ≥50 | 79 (30.3) | 182 (69.7) |  | 1.000 |
| **Marital Status** |  |  |  |  |
| Married | 124 (20.4) | 485 (79.6) | 25.766 (<0.001) | 0.747 (0.437 – 1.279) |
| Widowed | 111 (35.8) | 199 (64.2) |  | 1.319 (0.759 – 2.291) |
| Single | 25 (27.2) | 67 (72.8) |  | 1.000 |
| **Educational attainment of the respondents** |  |  |  |  |
| No formal education | 15 (31.3) | 33 (68.8) | 14.622 (0.001) | 1.565 (0.790 – 3.100) |
| Primary | 78 (35.0) | 145 (65.0) |  | 1.619 (1.128 – 2.325)* |
| Secondary and above | 167 (22.6) | 573 (77.4) |  | 1.000 |
| **Personal relationship with Health worker** |  |  |  |  |
| Yes | 73 (29.3) | 176 (70.7) | 2.241 (0.134) | 0.734 (0.520 – 1. 036) |
| No | 187 (24.5) | 575 (75.5) |  | 1.000 |
| **Household size** |  |  |  |  |
| <5 | 157 (28.9) | 386 (71.1) | 6.273 (0.012) | 1.204 (0.883 – 1.643) |
| ≥5 | 103 (22.0) | 365 (78.0) |  | 1.000 |
| **Household income per month (Naira)** |  |  |  |  |
| <50000 | 152 (22.9) | 513 (77.1) | 8.830 (0.012) | 0.491 (0.250 – 0.963)* |
| 50000 – 99000 | 92 (30.6) | 209 (69.4) |  | 0.706 (0.354 – 1.409) |
| ≥100000 | 16 (35.6) | 29 (64.4) |  | 1.000 |
| **Presence of other co-morbidities** |  |  |  |  |
| Yes | 47 (32.2) | 99 (67.8) | 6.843 (0.033) | 1.512 (0.721 – 3.170) |
| No | 196 (24.0) | 621 (76.0) |  | 0.761 (0.505 – 1.147) |
| I don’t know | 17 (35.4) | 31 (64.6) |  | 1.000 |
| **Social support** |  |  |  |  |
| Low support | 17 (41.5) | 24 (58.5) | 16.665 (<0.001) | 2.908 (1.396 – 6.059)* |
| Moderate | 200 (27.8) | 519 (72.2) |  | 1.898 (1.276 – 2.824)* |
| High | 43 (17.1) | 208 (82.9) |  | 1.000 |
| **Caregiver’s burden** |  |  |  |  |
| No – mild | 14 (15.1) | 79 (84.9) | 12.600 (0.002) | 0.688 (0.335 – 1.414) |
| Mild – moderate | 199 (28.9) | 489 (71.1) |  | 1.337 (0.899 – 1.989) |
| Severe | 47 (20.4) | 183 (79.6) |  | 1.000 |
