# Supplementary material for: “Carrying the burden silently”: A mixed-methods analysis of common mental health disorders among women caregivers living with HIV in Enugu, Nigeria
Source: PLOS Ment Health. 2026 Oct 23;3(10):e0000764. doi: 10.1371/journal.pmen.0000764 (PMC13693887; doi:10.1371/journal.pmen.0000764)
Supplement: Supplementary file 3 [file pmen.0000764.s004.docx]

**S2 Table: Multivariable logistic regression analysis for Anxiety disorder excluding perceived stress.**

| **Variables** | **(n=1011)**  **Anxiety disorder No anxiety disorder**  **N (%) N (%)** | | **χ2(p-value on**  **bivariate**  **analysis)** | **AOR in multivariate analysis (95% CI)** |
| --- | --- | --- | --- | --- |
| **Marital Status** |  |  |  |  |
| Married | 104 (17.1) | 505 (82.9) | 4.437 (0.109) | 0.758 (0.430 – 1.334) |
| Widowed | 70 (22.6) | 240 (77.4) |  | 1.132 (0.636 – 2.013) |
| Single | 20 (21.7) | 72 (78.3) |  | 1.000 |
| **Household size** |  |  |  |  |
| <5 | 114 (21.0) | 429 (79.0) | 2.466 (0.116) | 1.184 (0.848 – 1.655) |
| ≥5 | 80 (17.1) | 388 (82.9) |  | 1.000 |
| **Duration of living with HIV (months)** |  |  |  |  |
| <120 | 79 (22.2) | 277 (77.8) | 3.194 (0.074) | 2.002 (0.355 – 11.296) |
| ≥120 | 115 (17.6) | 540 (82.4) |  | 1.000 |
|  |  |  |  |  |
| **Duration on ART (months)** |  |  |  |  |
| <120 | 80 (21.9) | 285 (78.1) | 2.743 (0.098) | 0.599 (0.106 – 3.390) |
| ≥120 | 114 (17.6) | 532 (82.4) |  | 1.000 |
|  |  |  |  |  |
| **Viral load (copies/ml)** |  |  |  |  |
| <1000 | 185 (18.9) | 794 (81.1) | 1.702 (0.192) | 0.580 (0.261 – 1.290) |
| ≥1000 | 9 (28.1) | 23 (71.9) |  | 1.000 |
|  |  |  |  |  |
| **Presence of other co-morbidities** |  |  |  |  |
| Yes | 26 (17.8) | 120 (82.2) | 3.322 (0.190) | 2.027 (0.932 – 4..410) |
| No | 154 (18.8) | 663 (81.2) |  | 1.026 (0.639 – 1.647) |
| I don’t know | 14 (29.2) | 34 (70.8) |  | 1.000 |
| **Social support** |  |  |  |  |
| Low support | 12 (29.3) | 29 (70.7) | 6.332 (0.042) | 1.951 (0.878 – 4.332) |
| Moderate | 145 (20.2) | 574 (79.8) |  | 1.301 (0.854 – 1.983) |
| High | 37 (14.7) | 214 (85.3) |  | 1.000 |
| **Caregiver’s burden** |  |  |  |  |
| No – mild | 10 (10.8) | 83 (89.2) | 5.270 (0.072) | 0.412 (0.188 – 0.904)* |
| Mild – moderate | 134 (19.5) | 554 (80.5) |  | 0.768 (0.521 – 1.134) |
| Severe | 50 (21.7) | 180 (78.3) |  | 1.000 |
