# Supplementary material for: “Carrying the burden silently”: A mixed-methods analysis of common mental health disorders among women caregivers living with HIV in Enugu, Nigeria
Source: PLOS Ment Health. 2026 Oct 23;3(10):e0000764. doi: 10.1371/journal.pmen.0000764 (PMC13693887; doi:10.1371/journal.pmen.0000764)
Supplement: Supplementary file 4 [file pmen.0000764.s005.docx]

**S3 Table: Mixed-methods mapping of quantitative and qualitative findings**

| **Quantitative finding** | **Corresponding qualitative finding/theme** | **Mixed-methods interpretation** |
| --- | --- | --- |
| Low social support was associated with higher odds of depression (AOR = 2.32; 95% CI: 1.11–5.07). | Poor social support: Participants described emotional neglect, spousal mistreatment, isolation, and inadequate financial support as contributing to depression and difficulty fulfilling caregiving responsibilities. | Convergence: Both components indicate that inadequate social support is important to the psychological well-being of WCLHIV. |
| Lower perceived stress was associated with substantially lower odds of depression and anxiety. | Qualitative findings provided contextual examples of experiences that participants described as stressful or anxiety-provoking, including pill burden, concerns about medication availability, and fear of disclosure. | Complementarity: The qualitative findings provide contextual examples of circumstances that women experienced as stressful or anxiety-provoking. |
| Household income <₦50,000 was associated with lower odds of depression compared with the highest income category. | Cost: Participants described transportation costs, laboratory expenses, and limited financial resources as sources of psychological distress and depression. | Divergence: The qualitative accounts of financial hardship contrast with the inverse quantitative association between household income and depression. |
| Stigma was not significantly associated with depression in the quantitative regression model. | Stigma: Participants described discrimination and stigmatizing experiences from family members, healthcare workers, neighbours, and employers as contributing to sadness, isolation, and depression. | Divergence/complementarity: Although stigma was not independently associated with depression quantitatively, the qualitative findings demonstrate its perceived importance in participants’ lived experiences. |
| Primary education was associated with higher odds of depression compared with secondary education or above. | No specific qualitative theme directly examined educational attainment. | Quantitative-only finding: The qualitative component did not provide direct evidence to explain the association between educational attainment and depression. |
| Caregiver burden was not independently associated with anxiety in the primary model but became significant in the sensitivity analysis excluding perceived stress. | Some narratives described difficulty caring for children while dealing with inadequate support and personal psychological distress. | Partial complementarity: Qualitative accounts provide contextual evidence of caregiving difficulties, although caregiver burden did not emerge as a distinct qualitative theme. |
| No direct quantitative measure of pill burden or fear of disclosure was included as a predictor. | Pill burden/unavailability of drugs and fear of disclosure emerged as important qualitative themes associated with anxiety and depression. | Qualitative expansion: The qualitative component identified contextual influences on mental health that were not directly captured by the quantitative predictor variables. |

Note: Because different participants contributed to the quantitative and qualitative components, integration was undertaken at the level of overall findings and themes rather than through within-participant comparison.
