# Supplementary material for: “Carrying the burden silently”: A mixed-methods analysis of common mental health disorders among women caregivers living with HIV in Enugu, Nigeria
Source: PLOS Ment Health. 2026 Oct 23;3(10):e0000764. doi: 10.1371/journal.pmen.0000764 (PMC13693887; doi:10.1371/journal.pmen.0000764)
Supplement: Supplementary file 5 [file pmen.0000764.s006.docx]

STROBE Statement—Checklist of items that should be included in reports of ***cross-sectional studies***

|  | Item No | Recommendation | Pages and lines |
| --- | --- | --- | --- |
| **Title and abstract** | 1 | (*a*) Indicate the study’s design with a commonly used term in the title or the abstract | pg. 2, lines 1 & 38 |
|  |  | (*b*) Provide in the abstract an informative and balanced summary of what was done and what was found | pg. 2, lines 33–60 |
| Introduction | | |  |
| Background/rationale | 2 | Explain the scientific background and rationale for the investigation being reported | pg. 3–5, lines 69–121 |
| Objectives | 3 | State specific objectives, including any prespecified hypotheses | pg. 5, lines 120–122 |
| Methods | | |  |
| Study design | 4 | Present key elements of study design early in the paper | pg. 5–6, lines 134–154 |
| Setting | 5 | Describe the setting, locations, and relevant dates, including periods of recruitment, exposure, follow-up, and data collection | pg. 5–9, lines 123–211 |
| Participants | 6 | (*a*) Give the eligibility criteria, and the sources and methods of selection of participants | pg. 6, lines 141–154 |
| Variables | 7 | Clearly define all outcomes, exposures, predictors, potential confounders, and effect modifiers. Give diagnostic criteria, if applicable | pg. 7–9, lines 156–221; pg. 25–33 (Tables 1–5) |
| Data sources/ measurement | 8* | For each variable of interest, give sources of data and details of methods of assessment (measurement). Describe comparability of assessment methods if there is more than one group | pg. 7–8, lines 156–201 |
| Bias | 9 | Describe any efforts to address potential sources of bias | pg. 7, lines 159–174; pg. 21–22, lines 499–507 |
| Study size | 10 | Explain how the study size was arrived at | pg. 6, lines 147–152 |
| Quantitative variables | 11 | Explain how quantitative variables were handled in the analyses. If applicable, describe which groupings were chosen and why | pg. 8–9, lines 175–201; pp. 25–33 (Tables 1–5) |
| Statistical methods | 12 | (*a*) Describe all statistical methods, including those used to control for confounding | pg. 9, lines 213–221 |
|  |  | (*b*) Describe any methods used to examine subgroups and interactions | Not reported; no subgroup or interaction analyses are described. |
|  |  | (*c*) Explain how missing data were addressed | Not explicitly reported. Tables present the analysed sample, but missing-data handling is not described. |
|  |  | (*d*) If applicable, describe analytical methods taking account of sampling strategy | Not applicable as described; all eligible women during the study period were recruited (pg. 6, lines 149–154). |
|  |  | (*e*) Describe any sensitivity analyses | pg. 13, lines 281–295; S1–S2 Tables |
| Results | | |  |
| Participants | 13* | (a) Report numbers of individuals at each stage of study—eg numbers potentially eligible, examined for eligibility, confirmed eligible, included in the study, completing follow-up, and analysed | pg. 10–11, lines 242–248; Figure 1 |
|  |  | (b) Give reasons for non-participation at each stage | pg. 10, lines 242–245; Figure 1 |
|  |  | (c) Consider use of a flow diagram | pg. 11, lines 247–248; Figure 1 |
| Descriptive data | 14* | (a) Give characteristics of study participants (eg demographic, clinical, social) and information on exposures and potential confounders | pg. 11–12, lines 251–271; pp. 25–29 (Tables 1–3) |
|  |  | (b) Indicate number of participants with missing data for each variable of interest | Not explicitly reported. |
| Outcome data | 15* | Report numbers of outcome events or summary measures | pg. 12, lines 267–271; pp. 28–29 (Table 3) |
| Main results | 16 | (*a*) Give unadjusted estimates and, if applicable, confounder-adjusted estimates and their precision (eg, 95% confidence interval). Make clear which confounders were adjusted for and why they were included | pg. 12–13, lines 273–295; pp. 29–33 (Tables 4–5) |
|  |  | (*b*) Report category boundaries when continuous variables were categorized | pg. 7–8, lines 175–201; pp. 25–33 (Tables 1–5) |
|  |  | (*c*) If relevant, consider translating estimates of relative risk into absolute risk for a meaningful time period | Not applicable; odds ratios are reported for this cross-sectional analysis. |
| Other analyses | 17 | Report other analyses done—eg analyses of subgroups and interactions, and sensitivity analyses | pg. 13, lines 281–295; S1–S2 Tables |
| Discussion | | |  |
| Key results | 18 | Summarise key results with reference to study objectives | pg. 16–21, lines 373–486 |
| Limitations | 19 | Discuss limitations of the study, taking into account sources of potential bias or imprecision. Discuss both direction and magnitude of any potential bias | pg. 21–22, lines 487–507 |
| Interpretation | 20 | Give a cautious overall interpretation of results considering objectives, limitations, multiplicity of analyses, results from similar studies, and other relevant evidence | pg. 16–21, lines 373–486 |
| Generalisability | 21 | Discuss the generalisability (external validity) of the study results | pg. 17, lines 399–406; pg. 21–22, lines 494–498 |
| Other information | | |  |
| Funding | 22 | Give the source of funding and the role of the funders for the present study and, if applicable, for the original study on which the present article is based | Not reported in the manuscript. |

*Give information separately for exposed and unexposed groups.
