# Supplementary material for: “Carrying the burden silently”: A mixed-methods analysis of common mental health disorders among women caregivers living with HIV in Enugu, Nigeria
Source: PLOS Ment Health. 2026 Oct 23;3(10):e0000764. doi: 10.1371/journal.pmen.0000764 (PMC13693887; doi:10.1371/journal.pmen.0000764)
Supplement: Supplementary file 7 [file pmen.0000764.s009.docx]

**Journal Requirements**

**Comment:** Your manuscript is missing the following sections: Introduction. Please ensure these are present, and in the correct order, and that any references to subheadings in your main text are correct. An outline of the required sections can be consulted in our submission guidelines here: <https://journals.plos.org/mentalhealth/s/submission-guidelines#loc-parts-of-a-submission>.

**Response:** The introduction has been included. Thank you. Please see line 69

**Comment:** We have noticed that you have uploaded Supporting Information files, but you have not included a list of legends. Please add a full list of legends for your Supporting Information files after the references list.

**Response:** The full list of legends for the Supporting Information has been added after the reference section

**Comment:** We note that your Data Availability Statement is currently as follows: "The minimal data set is available as supporting information"
 
 Please confirm at this time whether or not your submission contains all raw data required to replicate the results of your study. Authors must share the “minimal data set” for their submission. PLOS defines the minimal data set to consist of the data required to replicate all study findings reported in the article, as well as related metadata and methods. <https://journals.plos.org/mentalhealth/s/#loc-minimal-data-set-definition>

**Response:** The submission contains quantitative and qualitative raw data required to replicate the results of our study. Please see S1 data and S2 Qualitative data

**Reviewer 1:** 

**Comment:** While the paper describes well the double burden, where women often have significant responsibilities that act as stressors in the context of living with HIV, it might be worth briefly summarising other ways that gender acts in this context. Do these work differently for women with and without additional caring responsibilities? ie to what extent is caring a unique additional burden, which is the premise of the paper?

**Response:** Thank you. This has been addressed in the introduction section. Please see lines 86 – 94

**Comment:** There is a very wide range of prevalence summarised in the introduction, but insufficient exploration of why this might be (methods, contexts, real findings??). This also raises the question of why this specific prevalence rate was chosen for the sample size calculation. Please explain.

**Response:** Thank you. We have revised the Introduction to clarify that the variation may reflect differences in study populations, geographical and sociocultural contexts, as well as methodological differences in the tools and approaches used to assess depression and anxiety across studies. Please see lines 82 – 85

Regarding why this specific prevalence rate was chosen for the sample size calculation, the prevalence of 22.7% used in the original sample-size calculation was obtained from a study among women living with HIV in a comparable sub-Saharan African clinical setting. It represented the proportion with comorbid anxiety and depression. We acknowledge that, since depression and anxiety were analysed as separate outcomes in the present study, calculating the required sample size separately for each outcome and selecting the larger estimate would have been a more conservative approach. This has been stated in the limitations section. Please see lines 490 – 494

**Comment:** While alluded to in the abstract, there is insufficient detail about the extent to which the measures used are valid for the cultural context and purpose of the research in the methods.

**Response:** Thank you. We have revised the Methods section to provide additional information on the cultural appropriateness and psychometric suitability of the measures used in the study. The questionnaire was pre-tested among women living with HIV in a comparable HIV clinic setting to assess clarity, comprehension, cultural appropriateness, and feasibility before the main data collection. We also added evidence on the prior validation, reliability, and psychometric performance of the individual instruments in Nigerian populations, as well as the Cronbach’s alpha coefficients obtained in our study. Please see lines 165 – 175

**Comment:** The exclusion of women who are on treatment for mental illness at the time of recruitment is problematic as would clearly change the prevalence findings - which is exactly the purpose of the study. Similarly, excluding people who are physically unwell means that a key factor in the determinants of mental health conditions (physical comorbidity) is removed from the findings. While there are clear practical reasons why these exclusions are routinely used, this is short-sighted and more effort can be made to gather this data) for example offering to try at a later date. These limitations should be described.

**Response:** Thank you. We agree that excluding women receiving treatment for a mental disorder and those who were too physically unwell to participate may have influenced the prevalence estimates and reduced representation of women with greater health needs. We have revised the limitations section to acknowledge the potential for underestimation of depression and anxiety arising from these exclusions. We also note that future studies should consider deferred recruitment of temporarily unwell participants to improve representativeness. Please see lines 495 – 499

**Comment:** Results: It was interesting that such a high proportion of participants were employed, as were their partners/husbands. It is worth commenting on how unusual this is, what the reason might be, and what effect it has on the results' generalisability.

**Response:** Thank you. The high employment rate likely reflects our broad definition of employment, which included informal and self-employed work in addition to formal employment. We have clarified this in the Discussion and noted that the predominance of economically active women may limit the generalisability of the findings to unemployed or more socioeconomically vulnerable women. Please see lines 400 – 406

**Comment:** It seemed odd that such a high number reported being on HRT, but only25% reported a relationship with a health worker.

**Response:** Thank you. The variable “relationship with a health worker” referred to whether the participant had a personal or family relationship with a healthcare worker, rather than routine contact with healthcare staff as part of HIV care. We agree that the original wording was ambiguous and have revised the table to read “personal relationship with a healthcare worker.” Please see Table 1

**Comment:** Discussion: Line 342-343. It is not accurate to categorically state that attribution for depression and anxiety is related to caring responsibility as the study methods do not allow this fully. There was not a comparison with non-career for example. It is more accurate to say that some of the women reported this burden as a factor.

**Response:** We have revised the Discussion to avoid attributing depression and anxiety directly to caregiving responsibilities. The text now reflects that some participants reported the combined demands of managing their own health while providing care and emotional support for their children as an additional source of psychological burden. Please see lines 382 – 384

**Comment:** It is worth noting that intersection between perceived stress and depression/anxiety in your interpretation. ie the women might be reporting the same thing, rather than your findings being that two independently identifies constructs were correlated.

**Response:** We agree that perceived stress may overlap conceptually with symptoms of depression and anxiety, such that these measures may partly capture related aspects of psychological distress. To examine whether inclusion of perceived stress influenced the observed associations, we excluded perceived stress from the multivariable regression models. In the depression model, primary education, lower household income, and low social support remained associated with depression, while in the anxiety model, caregiver burden became significant after perceived stress was removed. These findings suggest that some associations were independent of the perceived stress measure, although the conceptual overlap between perceived stress and depression/anxiety remains an important consideration and has been acknowledged in the Discussion. Please see lines 450 – 455 and lines 474 – 477

**Comment:** The limitations of the study are well described, but what might have been the effect of social desirability bias exactly? Would people have been reluctant to, or more likely to, disclose problems the clinicians/data collectors working in the service? What would implications of them doing so b perceived to be?

**Response:** We have revised the limitations section to clarify the likely direction and implications of social desirability bias. We now acknowledge that, because the questionnaire was interviewer-administered and some data collectors were healthcare workers in the study clinics, participants may have been reluctant to disclose psychological distress due to concerns about how such disclosures might be perceived by clinic staff or whether they could affect the care they received. This may have resulted in under-reporting of some psychosocial problems and consequently underestimated their prevalence. Please see lines 502 – 505

**Comment:** In addition, there are a number of minor typographical and grammatical errors.
Some examples are:
- Line 72: consistency in use of commas in number (also line 138). I think it is clearer with a comma, but the journal may have a style guidance on this.

**Response:** This has been addressed. Please see lines 72 & 152

**Comment:** - Line 113: Clinics should probably not be capitalized

**Response:** This has been addressed. Please see line 125

**Reviewer 2**

**Comment:** The exclusion criteria rule out women "on treatment for a mental disorder." That likely means some of the most affected women in this population never made it into the sample, which would push the depression and anxiety prevalence numbers down from what they'd actually be in the full population. This needs to be named as a limitation, it currently isn't, even though the authors do a good job flagging the cross-sectional design and interviewer-related bias elsewhere.

**Response:** We have revised the limitations section to acknowledge the potential for underestimation of depression and anxiety arising from the exclusion of women on treatment for mental disorders. Please see lines 495 – 499

**Comment:** None of the six tools used here (PHQ-9, GAD-7, PSS-10, HIV Stigma Scale, MSPSS, ZBI) were developed in Nigeria, and the paper doesn't report Cronbach's alpha for any of them in this sample, or say whether they were adapted or previously validated locally.

**Response:** We have reported evidence of their previous validation or use in Nigerian populations and included the Cronbach’s alpha coefficients obtained in our study. Please see lines 165 – 175

**Comment:** Given that some participants needed their consent form read aloud to them, I think it's fair to ask how comprehension of an English-language questionnaire was handled for women with limited literacy or English fluency.

**Response:** We have clarified how comprehension was addressed among participants with limited literacy or difficulty understanding English. The questionnaire was interviewer-administered, and where participants had difficulty understanding questions in English, trained research assistants explained the items in the participant’s preferred language while maintaining their intended meaning. Please see lines 158 – 161

**Comment:** Two of the tool citations don't check out. The ZBI is attributed to reference 18, which is word-for-word the same as reference 9 (the Adedoyin et al. Lagos caregiver study), not the actual source of the ZBI (Zarit, Reever and Bach-Peterson, 1980). And the PHQ-9 citation (Ford et al., 2020) is a paper about how clinicians use the PHQ-9 in practice, not the original validation study by Kroenke, Spitzer and Williams. Both need fixing.

**Response:** The tool citations for PHQ-9 and ZBI have been corrected. Please see references 20 & 25

**Comment:** The sample size math checks out (roughly 270 base sample, times a design effect of 3, times 1.1 for non-response, lands close to 900), but I'm not sure the design effect of 3 is justified given that recruitment was consecutive sampling across four facilities rather than an actual multi-stage cluster design.

**Response:** Thank you for pointing this obvious error. In reality, we recruited all eligible women who met the inclusion criteria within the study period. Please see lines 150 – 152

**Comment:** I'd also ask whether the regression models account for facility-level clustering at all, since if clustering was assumed to be strong enough to triple the required sample, it should show up somewhere in how the variance was estimated.

**Response:** As stated above, this was a total population sampling, and we didn’t apply clustering to the regression analysis

**Comment:** There's no participant flow reported anywhere, how many women were approached, how many declined, how many were excluded at each site. That's a fairly standard ask under STROBE for a cross-sectional study and would help readers judge how much selection bias might be at play.

**Response:** We have now added participant-flow information to the Results section and included a flow diagram. A total of 1,024 women were approached across the four study facilities; three were excluded because they did not meet the eligibility criteria, and 10 declined participation. The remaining 1,011 women were included in the final analysis. Please see lines 243 – 246 and Figure 1

**Comment:** Along the same lines, I didn't see a STROBE or COREQ checklist referenced for the quantitative and qualitative components respectively. PLOS Mental Health asks for these, so it's worth having the authors attach completed checklists as supporting information.

**Response:** We have completed and included the STROBE and COREQ checklists for the quantitative and qualitative components of the study respectively. Please see lines 140 - 144, as well as S4 and S5 tables

**Comment:** One more thing worth raising: perceived stress (PSS-10) is used as a predictor of both depression and anxiety, but stress and depressive or anxious symptoms overlap conceptually and often empirically. The size of the effects here, AOR of 0.05 for depression and 0.07 for anxiety, looks more consistent with that kind of overlap than with a genuinely independent predictor. I'd like to see the authors address this directly, or run the models again without stress in them to see what changes.

**Response:** Thank you. We agree that perceived stress, depression, and anxiety may overlap conceptually and empirically, and that the large effect estimates observed for perceived stress should therefore be interpreted cautiously. To address this concern, we reran the multivariable regression models excluding perceived stress. In the depression model, educational attainment, household income, and social support remained significantly associated with depression, with a somewhat stronger association observed for low social support. In the anxiety model, caregiver burden became significantly associated with anxiety. These analyses have been added to the Results and Supplementary Tables, and the Discussion has been revised to acknowledge the conceptual overlap between perceived stress and depressive/anxiety symptoms and to avoid interpreting perceived stress as a wholly independent predictor. Please see lines 450 – 455 and lines 474 – 477 in the Discussion section. Lines 282 – 286 and 293 – 295 for the Results section, as well as S1 and S2 tables

**Comment:** The income finding (lower income being protective against depression, contradicting the qualitative interviews) is already framed carefully as a possible explanation rather than a firm conclusion, which is the right call. I'd just ask the authors to say a bit more about what would actually need to be tested to confirm or rule out the explanation they're offering.

**Response:** We have added in the discussion section “future studies should assess broader measures of socioeconomic circumstances, including household wealth index, type of employment and household expenditure, to determine whether these factors confound or modify the observed association between household income and depression. Longitudinal studies would also be useful in clarifying the temporal relationship between changes in economic circumstances and depressive symptoms”. Please see lines 423 – 428

**Comment:** A few places in the discussion describe an AOR under 1 as "X times less likely," for instance "2.4 times less likely" for an AOR of 0.42. That's inverting an odds ratio as if it were a relative risk, and it's a common shorthand, but "58% lower odds" is more accurate and won't confuse readers who know the difference.
**Response:** We have corrected these to the appropriate percentages. Please see lines 412, 437, & 469

**Comment:** Table 3 lists the exact same standard deviation, 4.04, for both Perceived Stigma and Perceived Stress. These are different scales with different numbers of items, so I'd just want the authors to double check this wasn't copied over by mistake.

**Response:** We verified the calculations for perceived stigma and perceived stress in Table 3. The SDs were 4.04415 for perceived stigma and 4.03880 for perceived stress, both of which round to 4.04.

**Comment:** 98.3% of respondents are reported as employed, but 65.8% of households earn under ₦50,000 a month. Worth a line clarifying what counted as "employed" here, formal work versus any informal income-generating activity, since it changes how the income findings should be read.
**Response:** We have clarified in the Discussion that employment in this study included informal and self-employed income-generating activities, such as trading, farming, casual work, and artisanal activities, in addition to formal employment. We have also noted that the high employment rate did not necessarily indicate economic security, as most households reported relatively low monthly income. Please see lines 401 – 407

**Comment:** Small typo around line 134: "formular" should be "formula."

**Response:** This has been corrected. Please see line 148

**Comment:** Neither logistic regression model reports any diagnostics, goodness of fit, multicollinearity, pseudo-R², anything that would let a reader judge how well the models actually fit the data.

**Response:** We have now added model-fit information for both multivariable logistic regression models. Goodness of fit was assessed using the Hosmer–Lemeshow test, and model explanatory ability was assessed using the Nagelkerke R². The depression model showed no evidence of poor fit (Hosmer–Lemeshow χ² = 11.70, df = 8, p = 0.165) and had a Nagelkerke R² of 0.157. Similarly, the anxiety model showed no evidence of poor fit (Hosmer–Lemeshow χ² = 5.11, df = 8, p = 0.746) and had a Nagelkerke R² of 0.079. These results have been added to the Results section. Please see lines 280 – 281 & 291 – 292

**Comment:** The mixed-methods integration is one of the paper's real strengths, but the quantitative and qualitative samples were entirely different women, which is a reasonable way to avoid respondent fatigue, but it does mean the "convergence" here is happening at the level of themes rather than the same individuals. Worth stating that plainly, and a table mapping the quantitative predictors onto the qualitative themes side by side would make the integration much easier to follow.

**Response:** We have stated in the Methods that different participants contributed to the quantitative and qualitative components of the study. Accordingly, integration occurred at the level of themes rather than through within-participant comparison. Please see lines 138 – 140.
We have also included in S3 a table that maps the key quantitative findings of the predictors alongside the corresponding qualitative themes to make areas of convergence, complementarity, and divergence more explicit. Please see S3 table

**Comment:** Data availability is fine, the minimal dataset is included as supporting information. Ethics approval and consent procedures, including accommodations for participants who couldn't read or write, are handled properly, and I have no concerns about dual publication or research integrity. The writing itself is clear and standard English throughout; the only language-related notes are the typo and the odds ratio phrasing above.

**Response:** Thank you. The typos and odds ratio have been corrected. Please see lines 148, 412, 437, & 469.
