# Supplementary material for: “Carrying the burden silently”: A mixed-methods analysis of common mental health disorders among women caregivers living with HIV in Enugu, Nigeria
Source: PLOS Ment Health. 2026 Oct 23;3(10):e0000764. doi: 10.1371/journal.pmen.0000764 (PMC13693887; doi:10.1371/journal.pmen.0000764)
Supplement: Peer review history

# Peer review: Decision Letter 0

**DOI:** 10.1371/journal.pmen.0000764.r001

**Authors:** Anil Fastenau

31 Aug 2026

PMEN-D-26-00314

“Carrying the burden silently”: A mixed-methods analysis of common mental health disorders among women caregivers living with HIV in Enugu, Nigeria

PLOS Mental Health

Dear Dr. Ezenwosu,

Thank you for submitting your manuscript to PLOS Mental Health. After careful consideration, we feel that it has merit but does not fully meet PLOS Mental Health’s publication criteria as it currently stands. Therefore, we invite you to submit a revised version of the manuscript that addresses the points raised during the review process.

Please submit your revised manuscript by Sep 30 2026 11:59PM. If you will need more time than this to complete your revisions, please reply to this message or contact the journal office at mentalhealth@plos.org. When you're ready to submit your revision, log on to https://www.editorialmanager.com/pmen/ and select the 'Submissions Needing Revision' folder to locate your manuscript file.

Please include the following items when submitting your revised manuscript:

- A letter that responds to each point raised by the editor and reviewer(s). You should upload this letter as a separate file labeled 'Response to Reviewers'.
- A marked-up copy of your manuscript that highlights changes made to the original version. You should upload this as a separate file labeled 'Revised Manuscript with Track Changes'.
- An unmarked version of your revised paper without tracked changes. You should upload this as a separate file labeled 'Manuscript'.

Guidelines for resubmitting your figure files are available below the reviewer comments at the end of this letter.

As the corresponding author, your ORCID iD is verified in the submission system and will appear in the published article. PLOS supports the use of ORCID, and we encourage all coauthors to register for an ORCID iD and use it as well. Please encourage your coauthors to verify their ORCID iD within the submission system before final acceptance, as unverified ORCID iDs will not appear in the published article. *Only*  the individual author can complete the verification step; PLOS staff *cannot*  verify ORCID iDs on behalf of authors.

We look forward to receiving your revised manuscript.

Kind regards,

Anil Fastenau, M.D., M.Sc.

Academic Editor

PLOS Mental Health

Journal Requirements:

1. Your manuscript is missing the following sections: Introduction. Please ensure these are present, and in the correct order, and that any references to subheadings in your main text are correct. An outline of the required sections can be consulted in our submission guidelines here: https://journals.plos.org/mentalhealth/s/submission-guidelines#loc-parts-of-a-submission.

2. We have noticed that you have uploaded Supporting Information files, but you have not included a list of legends. Please add a full list of legends for your Supporting Information files after the references list.

3. We note that your Data Availability Statement is currently as follows: "The minimal data set is available as supporting information"

Please confirm at this time whether or not your submission contains all raw data required to replicate the results of your study. Authors must share the “minimal data set” for their submission. PLOS defines the minimal data set to consist of the data required to replicate all study findings reported in the article, as well as related metadata and methods.

https://journals.plos.org/mentalhealth/s/#loc-minimal-data-set-definition

For example, authors should submit the following data:

- The values behind the means, standard deviations and other measures reported;

- The values used to build graphs;

- The points extracted from images for analysis.

Authors do not need to submit their entire data set if only a portion of the data was used in the reported study.

If your submission does not contain these data, please either upload them as Supporting Information files or deposit them to a stable, public repository and provide us with the relevant URLs, DOIs, or accession numbers. For a list of recommended repositories, please see:

https://journals.plos.org/mentalhealth/s/recommended-repositories

If there are ethical or legal restrictions on sharing a de-identified data set, please explain them in detail (e.g., data contain potentially sensitive information, data are owned by a third-party organization, etc.) and who has imposed them (e.g., an ethics committee). Please also provide contact information for a data access committee, ethics committee, or other institutional body to which data requests may be sent. If data are owned by a third party, please indicate how others may request data access.

If the reviewer comments include a recommendation to cite specific previously published works, please review and evaluate these publications to determine whether they are relevant and should be cited. There is no requirement to cite these works unless the editor has indicated otherwise.

Please review your reference list to ensure that it is complete and correct. If you have cited papers that have been retracted, please include the rationale for doing so in the manuscript text, or remove these references and replace them with relevant current references. Any changes to the reference list should be mentioned in the rebuttal letter that accompanies your revised manuscript. If you need to cite a retracted article, indicate the article’s retracted status in the References list and also include a citation and full reference for the retraction notice.

Additional Editor Comments (if provided):

[Note: HTML markup is below. Please do not edit.]

Reviewers' comments:

Reviewer's Responses to Questions

**Comments to the Author**

1. Does this manuscript meet PLOS Mental Health’s publication criteria?

Reviewer #1: Yes

Reviewer #2: Partly

**********

2. Has the statistical analysis been performed appropriately and rigorously?

Reviewer #1: Yes

Reviewer #2: Yes

**********

3. Have the authors made all data underlying the findings in their manuscript fully available (please refer to the Data Availability Statement at the start of the manuscript PDF file)?

The PLOS Data policy

Reviewer #1: Yes

Reviewer #2: Yes

**********

4. Is the manuscript presented in an intelligible fashion and written in standard English?

Reviewer #1: Yes

Reviewer #2: Yes

**********

**Review Comments to the Author**

**Reviewer #1:**  This article is a useful contribution to the literature exploring the experience of women living with HIV.

It is methodologically strong, and very well written.

I have several mainly minor comments:

1. While the paper describes well the double burden, where women often have significant responsibilities that act as stressors in the context of living with HIV, it might be worth briefly summarising other ways that gender acts in this context. Do these work differently for women with and without additional caring responsibilities? ie to what extent is caring a unique additional burden, which is the premise of the paper?

2. There is a very wide range of prevalence summarised in the introduction, but insufficient exploration of why this might be (methods, contexts, real findings??). This also raises the question of why this specific prevalence rate was chosen for the sample size calculation. Please explain.

3. While alluded to in the abstract, there is insufficient detail about the extent to which the measures used are valid for the cultural context and purpose of the research in the methods.

4. The exclusion of women who are on treatment for mental illness at the time of recruitment is problematic as would clearly change the prevalence findings - which is exactly the purpose of the study. Similarly, excluding people who are physically unwell means that a key factor in the determinants of mental health conditions (physical comorbidity) is removed from the findings. While there are clear practical reasons why these exclusions are routinely used, this is short-sighted and more effort can be made to gather this data) for example offering to try at a later date. These limitations should be described.

5. Results: It was interesting that such a high proportion of participants were employed, as were their partners/husbands. It is worth commenting on how unusual this is, what the reason might be, and what effect it has on the results' generalisability.

It seemed odd that such a high number reported being on HRT, but only25% reported a relationship with a health workers.

6. Discussion: Line 342-343. It is not accurate to categorically state that attribution for depression and anxiety is related to caring responsibility as the study methods do not allow this fully. There was not a comparison with non-career for example. It is more accurate to say that some of the women reported this burden as a factor.

It is worth noting that intersection between perceived stress and depression/anxiety in your interpretation. ie the women might be reporting the same thing, rather than your findings being that two independently identifies constructs were correlated.

7. The limitations of the study are well described, but what might have been the effect of social desirability bias exactly? Would people have been reluctant to, or more likely to, disclose problems the clinicians/data collectors working in the service? What would implications of them doing so b perceived to be?

In addition, there are a number of minor typographical and grammatical errors.

Some examples are:

- Line 72: consistency in use of commas in number (also line 138). I think it is clearer with a comma, but the journal may have a style guidance on this.

- Line 113: Clinics should probably not be capitalised

Reviewer #2: Summary and recommendation

This is a solid, well-executed study on a population that genuinely doesn't get enough attention in the literature, women caregivers living with HIV, as distinct from women living with HIV more broadly. The design is sound, the ethics are handled properly, and the quantitative and qualitative findings mostly talk to each other in a way that strengthens the paper. I'd recommend minor revision. My comments below cover what I think needs addressing before this is ready.

Major points

The exclusion criteria rule out women "on treatment for a mental disorder." That likely means some of the most affected women in this population never made it into the sample, which would push the depression and anxiety prevalence numbers down from what they'd actually be in the full population. This needs to be named as a limitation, it currently isn't, even though the authors do a good job flagging the cross-sectional design and interviewer-related bias elsewhere.

None of the six tools used here (PHQ-9, GAD-7, PSS-10, HIV Stigma Scale, MSPSS, ZBI) were developed in Nigeria, and the paper doesn't report Cronbach's alpha for any of them in this sample, or say whether they were adapted or previously validated locally. Given that some participants needed their consent form read aloud to them, I think it's fair to ask how comprehension of an English-language questionnaire was handled for women with limited literacy or English fluency.

Two of the tool citations don't check out. The ZBI is attributed to reference 18, which is word-for-word the same as reference 9 (the Adedoyin et al. Lagos caregiver study), not the actual source of the ZBI (Zarit, Reever and Bach-Peterson, 1980). And the PHQ-9 citation (Ford et al., 2020) is a paper about how clinicians use the PHQ-9 in practice, not the original validation study by Kroenke, Spitzer and Williams. Both need fixing.

The sample size math checks out (roughly 270 base sample, times a design effect of 3, times 1.1 for non-response, lands close to 900), but I'm not sure the design effect of 3 is justified given that recruitment was consecutive sampling across four facilities rather than an actual multi-stage cluster design. I'd also ask whether the regression models account for facility-level clustering at all, since if clustering was assumed to be strong enough to triple the required sample, it should show up somewhere in how the variance was estimated.

There's no participant flow reported anywhere, how many women were approached, how many declined, how many were excluded at each site. That's a fairly standard ask under STROBE for a cross-sectional study and would help readers judge how much selection bias might be at play.

Along the same lines, I didn't see a STROBE or COREQ checklist referenced for the quantitative and qualitative components respectively. PLOS Mental Health asks for these, so it's worth having the authors attach completed checklists as supporting information.

One more thing worth raising: perceived stress (PSS-10) is used as a predictor of both depression and anxiety, but stress and depressive or anxious symptoms overlap conceptually and often empirically. The size of the effects here, AOR of 0.05 for depression and 0.07 for anxiety, looks more consistent with that kind of overlap than with a genuinely independent predictor. I'd like to see the authors address this directly, or run the models again without stress in them to see what changes.

Smaller things

The income finding (lower income being protective against depression, contradicting the qualitative interviews) is already framed carefully as a possible explanation rather than a firm conclusion, which is the right call. I'd just ask the authors to say a bit more about what would actually need to be tested to confirm or rule out the explanation they're offering.

A few places in the discussion describe an AOR under 1 as "X times less likely," for instance "2.4 times less likely" for an AOR of 0.42. That's inverting an odds ratio as if it were a relative risk, and it's a common shorthand, but "58% lower odds" is more accurate and won't confuse readers who know the difference.

Table 3 lists the exact same standard deviation, 4.04, for both Perceived Stigma and Perceived Stress. These are different scales with different numbers of items, so I'd just want the authors to double check this wasn't copied over by mistake.

98.3% of respondents are reported as employed, but 65.8% of households earn under ₦50,000 a month. Worth a line clarifying what counted as "employed" here, formal work versus any informal income-generating activity, since it changes how the income findings should be read.

Small typo around line 134: "formular" should be "formula."

Neither logistic regression model reports any diagnostics, goodness of fit, multicollinearity, pseudo-R², anything that would let a reader judge how well the models actually fit the data.

The mixed-methods integration is one of the paper's real strengths, but the quantitative and qualitative samples were entirely different women, which is a reasonable way to avoid respondent fatigue, but it does mean the "convergence" here is happening at the level of themes rather than the same individuals. Worth stating that plainly, and a table mapping the quantitative predictors onto the qualitative themes side by side would make the integration much easier to follow.

Data, ethics and language

Data availability is fine, the minimal dataset is included as supporting information. Ethics approval and consent procedures, including accommodations for participants who couldn't read or write, are handled properly, and I have no concerns about dual publication or research integrity. The writing itself is clear and standard English throughout; the only language-related notes are the typo and the odds ratio phrasing above.

**********

what does this mean?). If published, this will include your full peer review and any attached files.

**Do you want your identity to be public for this peer review?**  If you choose “no”, your identity will remain anonymous but your review may still be made public.

For information about this choice, including consent withdrawal, please see our Privacy Policy

Reviewer #1: No

Reviewer #2: **Yes:**  Christine Iyenrhe Bestman

**********

[NOTE: If reviewer comments were submitted as an attachment file, they will be attached to this email and accessible via the submission site. Please log into your account, locate the manuscript record, and check for the action link "View Attachments". If this link does not appear, there are no attachment files.]

# Author comment: Author response to Decision Letter 1

**DOI:** 10.1371/journal.pmen.0000764.r002

30 Sep 2026

Attachment Submitted filename: Response to reviewers.docx

# Editor report: Decision Letter 1

**DOI:** 10.1371/journal.pmen.0000764.r003

**Authors:** Anil Fastenau

7 Oct 2026

“Carrying the burden silently”: A mixed-methods analysis of common mental health disorders among women caregivers living with HIV in Enugu, Nigeria

PMEN-D-26-00314R1

Dear Dr Ezenwosu,

We are pleased to inform you that your manuscript '“Carrying the burden silently”: A mixed-methods analysis of common mental health disorders among women caregivers living with HIV in Enugu, Nigeria' has been provisionally accepted for publication in PLOS Mental Health.

Before your manuscript can be formally accepted you will need to complete some formatting changes, which you will receive in a follow up email. A member of our team will be in touch with a set of requests.

Please note that your manuscript will not be scheduled for publication until you have made the required changes, so a swift response is appreciated.

IMPORTANT: The editorial review process is now complete. PLOS will only permit corrections to spelling, formatting or significant scientific errors from this point onwards. Requests for major changes, or any which affect the scientific understanding of your work, will cause delays to the publication date of your manuscript.

If your institution or institutions have a press office, please notify them about your upcoming paper to help maximize its impact. If they'll be preparing press materials, please inform our press team as soon as possible -- no later than 48 hours after receiving the formal acceptance. Your manuscript will remain under strict press embargo until 2 pm Eastern Time on the date of publication. For more information, please contact mentalhealth@plos.org.

Thank you again for supporting Open Access publishing; we are looking forward to publishing your work in PLOS Mental Health.

Best regards,

Anil Fastenau, M.D., M.Sc.

Academic Editor

PLOS Mental Health

***********************************************************

Reviewer Comments (if any, and for reference):
